# The enantioselective synthesis of (*S*)-(+)-mianserin and (*S*)-(+)-epinastine

**DOI:** 10.3762/bjoc.11.164

**Published:** 2015-08-28

**Authors:** Piotr Roszkowski, Jan K Maurin, Zbigniew Czarnocki

**Affiliations:** 1 Faculty of Chemistry, University of Warsaw, Pasteura 1, 02-093, Warsaw, Poland; 2 National Medicines Institute, Chełmska 30/34, 00-725 Warsaw, Poland and; 3 National Centre for Nuclear Research, Andrzeja Sołtana 7, 05-400 Otwock-Świerk, Poland

**Keywords:** chiral diamines, enantioselective reduction, epinastine, mianserin, ruthenium complexes

## Abstract

A simple enantioselective synthetic procedure for the preparation of mianserin and epinastine in optically pure form is described. The key step in the synthetic pathway is the asymmetric reduction of the cyclic imine using asymmetric transfer hydrogenation conditions.

## Introduction

**Figure 1:**
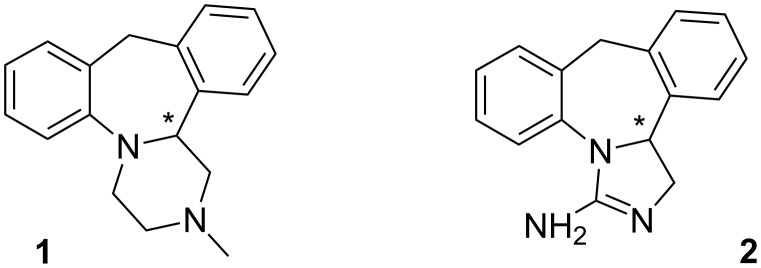
The structure of mianserin **1** and epinastine **2**.

Mianserin (**1**) is a tetracyclic compound widely used as a drug in the treatment of depression. Despite the fact that the (*S*)-(+)-enantiomer of mianserin is more potent than the (*R*)-antipode in pharmacological tests for antidepresant activity [1–3], it is still administered as a racemate probably due to the fact that no serious adverse effects have been recorded for the (*R*)-enantiomer. Interestingly, no enantioselective synthesis of mianserin has been developed so far. The synthesis of racemic mianserin was originally described by Organon [4–5], and in 1999 Jackson and Subasinghe presented a method for the separation of mianserin enantiomers with (+)- or (−)-di-*p*-toluoyltartaric acid [6]. Later, the diastereoselective synthesis of (*R*)-mianserin using (*S*)-(−)-α-methylbenzylamine as a chiral auxiliary was described by the Czarnocki group [7]. For the commercial manufacture of mianserin enantiomers both the above mentioned processes are rather unjustified economically and the most advantageous method could be the enantioselective synthesis. Considering our experience in asymmetric synthesis of structurally similar aptazepine [8], we applied this protocol to the preparation of (*S*)-mianserin. The key step in the proposed synthetic pathway is the enantioselective reduction of an endocyclic imine that led to the chiral amine. Interestingly, starting from this intermediate we obtained mianserin and also another important active substance, epinastine, in enantiomerically pure form (Figure 1).

Epinastine (**2**) is a histamine H_1_ receptor antagonist and is used as racemic mixture in eye drops to relieve the symptoms of allergic conjunctivitis. Analogously as in the case of mianserin, the (*S*)-enantiomer is the more active form [9].

In a key step in the enantioselective synthesis of mianserin and epinastine we applied the asymmetric reduction of the prochiral imine by asymmetric hydrogen transfer reaction (ATH) [10–14]. The proposed strategy could be used for the preparation of the title compounds and their structural analogues.

## Results and Discussion

**Scheme 1:**
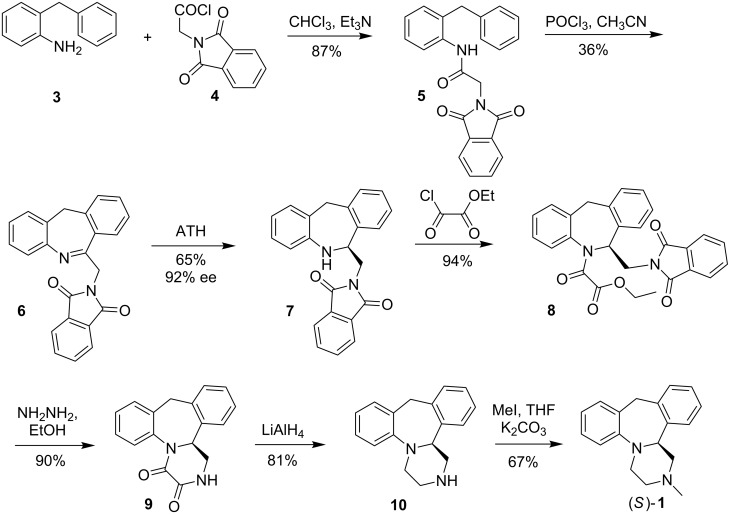
Enantioselective synthesis of (*S*)-(+)-mianserin.

The synthetic pathway is presented in Scheme 1. The reaction of 2-benzylaniline (**3**) with *N*-phthalylglycyl chloride (**4**) gave amide **5** in 87% yield. The Bischler–Napieralski condensation of amide **5** using phosphorus oxychloride in acetonitrile led to endocyclic imine **6** in 36% yield. The unreacted compound **5** could be fully recycled.

When the cyclization was carried out in toluene or without the solvent, only traces of the imine were detected. Alternatively the condensation of *N*-chloroacetyl-2-benzylaniline and subsequent reaction with potassium phthalimide proposed by Moffett [15] may be used for this step with comparable results. The imine **6** was then transformed to the enantiomerically enriched amine **7** with the aid of asymmetric transfer hydrogenation (ATH) process [8,10–11].

**Figure 2:**
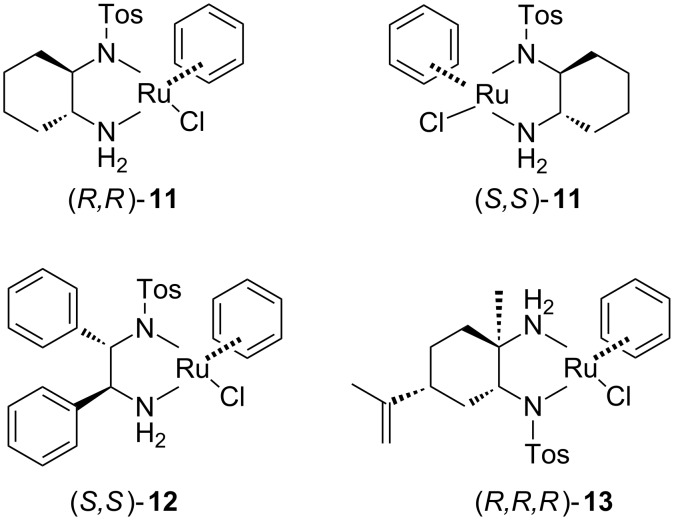
Catalysts used in ATH.

As in our synthesis of aptazepine [8], we initially used the chiral ruthenium complex **11** which contain (1*R*,2*R*)- or (1*S*,2*S*)-*N*-tosyl-1,2-cyclohexanediamine as chiral ligand (Figure 2).

The reaction was carried out in acetonitrile using an azeotropic mixture of formic acid/triethylamine as hydrogen source with 50:1 substrate to catalyst ratio. Under these conditions the desired amine was obtained in only 11% yield and with an enantioselectivity of 60% ee (Table 1). The low chemical yield probably results from the limited solubility of the imine in acetonitrile. When dichloromethane was used as a solvent, the chemical yield increased to 65% and the stereoselectivity achieved 92% ee (Table 1, entries 2 and 3). Additionally, chloroform and dimethylformamide were tested as solvents. The reduction in chloroform gave worse results (45% yield and 73% ee) than the reaction in dichloromethane, but the hydrogenation of imine **6** in DMF using (*R*,*R*)-**11** gave the product with the highest enantiomeric excess 95%, but with only 26% chemical yield (Table 1, entry 6).

**Table 1:** The asymmetric reduction imine **6** by asymmetric transfer hydrogenation^a^.

Entry	Catalyst	Solvent	Time (h)	Yield (%)	[α]_D_^23^	ee (%)^b^, ()^c^

1	(*R*,*R*)-**11**	CH_3_CN	72	11	+41.0	60 (*S*)
2	(*R*,*R*)-**11**	CH_2_Cl_2_	24	65	+62.8	92 (*S*)
3	(*S*,*S*)-**11**	CH_2_Cl_2_	24	63	−62.2	91 (*R*)
4	(*R*,*R*)-**11**	CHCl_3_	24	45	+50.1	73 (*S*)
5	(*S*,*S*)-**11**	CHCl_3_	24	47	−49.3	72 (*R*)
6	(*R*,*R*)-**11**	DMF	24	26	+65.0	95 (*S*)
7	(*S*,*S*)-**12**	CH_3_CN	74	0	−	−
8	(*S*,*S*)-**12**	CH_2_Cl_2_	24	51	−49.0	72 (*R*)
9	(*S*,*S*)-**12**	CHCl_3_	24	56	−50.3	73 (*R*)
10	(*S*,*S*)-**12**	DMF	24	23	−50.1	73 (*R*)
11	(*R*,*R,R*)-**13**	CH_2_Cl_2_	24	52	+51.6	75 (*S*)
12	(*R*,*R,R*)-**13**	DMF	24	21	+42.1	62 (*S*)

^a^The reaction was carried out at 22–24 °C using imine **6** (0.284 mmol) in solvent (5 mL) and a formic acid/triethylamine mixture (5:2, 1 mL) with a substrate to catalyst ratio S/C = 50. ^b^Determined by the value of the specific rotation of the isolated product. ^c^Determined by the comparison with X-ray data.

Subsequently other catalysts were tested in order to improve the efficiency of the reduction. The catalyst **12**, which is based on *N*-tosyl-(1*S*,2*S*)-1,2-diphenylethane-1,2-diamine (TsDPEN), gave amine **7** with 72–73% ee regardless of the solvent used, with the exception of acetonitrile (Table 1, entries 8–10). In CH_3_CN this catalyst is inactive and the product was not formed. The solvent effect on the chemical yield was similar to that observed with catalyst **11** (Table 1). Finally, the ruthenium complex **13** modified with amine developed by us [16] was tested. This catalyst possesses similar activity to catalyst **12** and gave the product with 75% enantiomeric excess and 52% yield. Applying dimethylformamide as a solvent led to lower asymmetric induction and to significant reduction of the yield to 21%.

**Figure 3:**
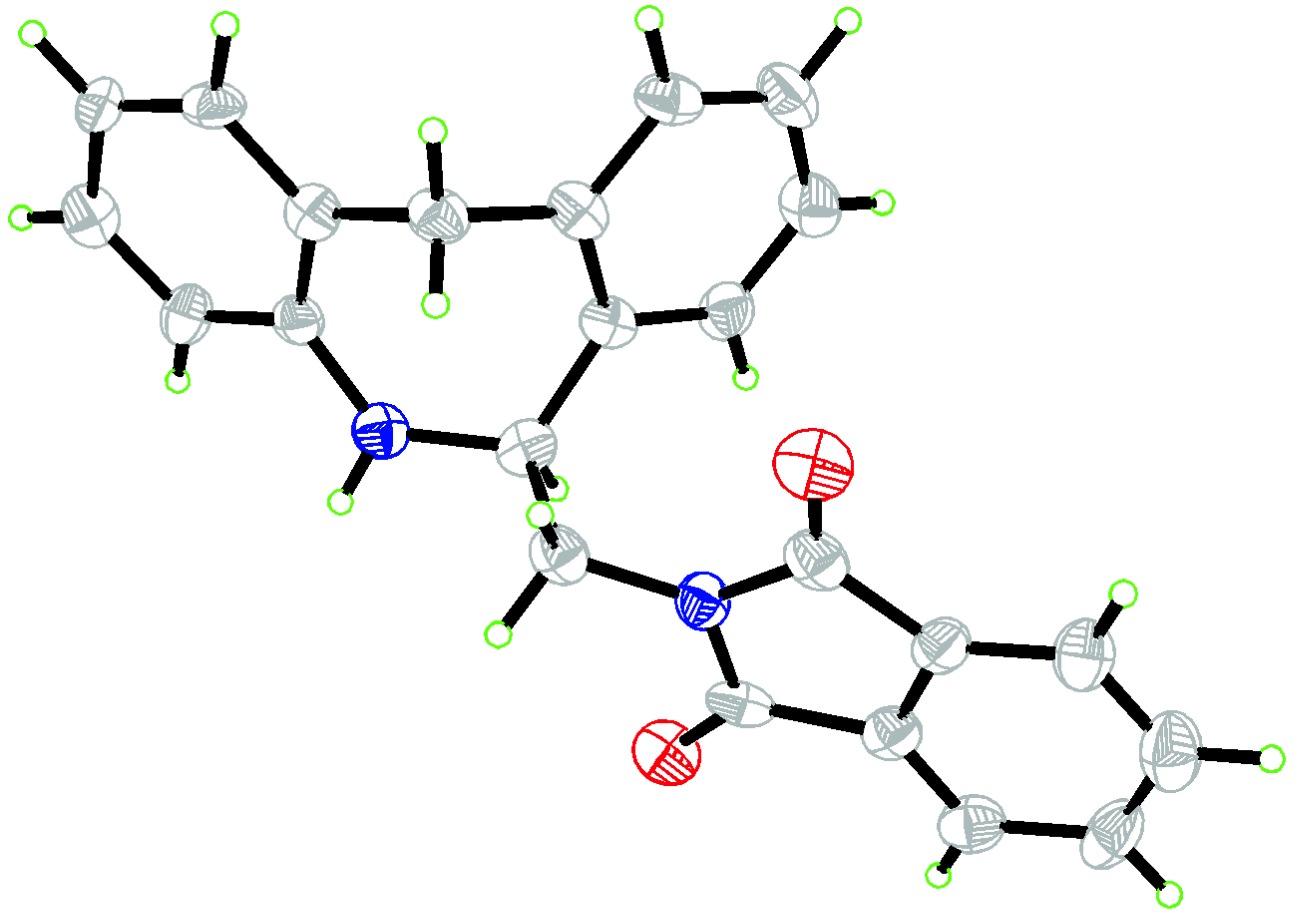
The ORTEP diagram for X-ray analysis of compound (*S*)-**7**.

The recrystallization of amine **7** (60–95% ee) from a chloroform/methanol/diethyl ether mixture gave this compound with 46–91% recovery of the pure enantiomer. A single recrystallization is sufficient for the amine enantioenriched above 75% but in the case of the amine with a lower optical purity (60–75%) a double recrystallization was applied. The values of specific rotation were [α]_D_^23^ +68.1 (*c* 1, CHCl_3_) and [α]_D_^23^ −68.3 (*c* 1, CHCl_3_), which could not be changed by repeated crystallization steps. Confirmation of the optical purity of compound **7** by HPLC (Daicel OD-H) could not be obtained because of its insufficient solubility in a hexane/2-propanol mixture. The absolute configuration of amine (*S*)-**7** obtained by reduction of **6** using (*R*,*R*)-**11** as catalyst was confirmed by X-ray analysis (Figure 3).

Subsequently, the synthetic procedure developed by us for aptazepine was applied [8]. Amide **8** was obtained in 94% yield by reacting amine **7** and ethyl chloro(oxo)acetate in dichloromethane in the presence of triethylamine. The removing of the phthaloyl protecting group from **8** using hydrazine gave an intermediate with a free amino group which is cyclised in situ to dioxopiperazine **9** in 90% yield. In the next step, desmethylmianserin (**10**) was obtained in 81% yield by reduction of the carbonyl groups using a 1.0 M solution of lithium aluminum hydride in THF. Finally, the reaction between derivative **10** and methyl iodide led to optical pure (*S*)-(+)-mianserin (**1**) (as confirmed by chiral HPLC) in 67% yield.

In order to obtain standards for HPLC analysis, racemic derivatives **7–10** and **1** were made (Scheme 1). Compounds **7**–**9** were insoluble in hexane/2-propanol mixture, a typical mobile phase for HPLC analysis on an OD-H column. Therefore chromatographic analysis was done for amines **10** and **1** only. For mianserin satisfactory resolution conditions were found that confirmed the optical purity of the final product (*S*)-**1** [17].

**Scheme 2:**
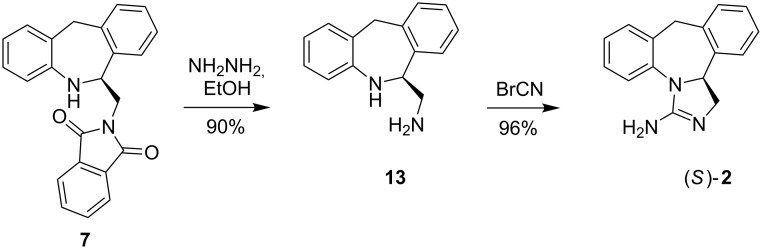
Enantioselective synthesis of (*S*)-(+)-epinastine.

The preparation of optically pure amine **7** allows also for its transformation to epinastine (**2**). The synthesis is presented in Scheme 2 and is based on the modified procedure described by Schneider [18]. The reaction of (*S*)-(+)-**7** with hydrazine led to derivative **13** with deprotected amine function. Subsequent condensation with cyanogen bromide gave (*S*)-(+)-epinastine (**2**) in nearly quantitative yield.

## Conclusion

In summary, we have presented a simple enantioselective synthesis of (*S*)-(+)-mianserin and (*S)*-(+)-epinastine. Chirality was introduced in a key step by asymmetric transfer hydrogenation. This synthetic procedure could be used for the preparation of other compounds variously substituted at the aryl rings.

## Supporting Information

File 1 Experimental procedures, spectroscopic and analytical data, and copies of NMR spectra for all described compounds.
