# Supplementary material for: The enantioselective synthesis of (S)-(+)-mianserin and (S)-(+)-epinastine
Source: Beilstein J Org Chem. 2015 Aug 28;11:1509–13. doi: 10.3762/bjoc.11.164 (PMC4578372; doi:10.3762/bjoc.11.164)
Supplement: File 1 — Experimental procedures, spectroscopic and analytical data, and copies of NMR spectra for all described compounds. [file Beilstein_J_Org_Chem-11-1509-s001.pdf]

Supporting Information

for

The enantioselective synthesis of (*S*)-(+)-mianserin and (*S*)-(+)-epinastine

Piotr Roszkowski*[1], Jan. K. Maurin[2,3] and Zbigniew Czarnocki[1]

Address: [1]Faculty of Chemistry, University of Warsaw, Pasteura 1, 02-093, Warsaw, Poland, [2] National Medicines Institute, Chełmska 30/34, 00-725 Warsaw, Poland and [3]National Centre for Nuclear Research, Andrzeja Sołtana 7, 05-400 Otwock-Świerk, Poland

Email: Piotr Roszkowski* - roszkowski@chem.uw.edu.pl

* Corresponding author

Experimental procedures, spectroscopic and analytical data, and copies of NMR spectra for all described compounds

General Methods

All reagents were purchased from commercial suppliers and were used without further purification. The NMR spectra were recorded on a Bruker AVANCE spectrometer operating at 500 MHz or 300 MHz for ^{1}H NMR and at 125 MHz or 75 MHz for ^{13}C NMR. The spectra were measured in $CDCl_3$ and are given as δ values (in ppm) relative to TMS. Mass spectra were collected on a Quatro LC Micromass and a LCT Micromass TOF HiRes apparatus. The IR spectra were recorded using Shimadzu FTIR-8400S spectrometer. Optical rotation was measured on a Perkin-Elmer 247 MC polarimeter. TLC analyses were performed on silica gel plates (Merck Kiesegel GF_{254}) and visualized using UV light or iodine vapour. Column chromatography was carried out at atmospheric pressure using Silica Gel 60 (230–400 mesh, Merck) or aluminium oxide and using chloroform or dichloromethane or mixtures of chloroform/methanol. The optical purity was determined by HPLC analysis using Chiralcel OD-H column. Melting points were determined on a Melting Point Meter KSP1D and were uncorrected. All solvents used in the reactions were anhydrous. The single crystal X-ray measurements were performed on an Oxford Diffraction Excalibur R CCD κ-axis diffractometer using monochromatic Cu Kα radiation. After initial corrections and data reduction, intensities of reflections were used to solve and consecutively refine structures using SHELXS97 and SHELXL97 programs [1].

***N*-(2-Benzylphenyl)-2-(1,3-dioxo-1,3-dihydroisoindol-2-yl)acetamide (5)**

To a stirred solution of **3** (10.00 g, 54.57 mmol) and triethylamine (15.21 mL, 109.10 mmol) in 200 mL $CHCl_3$ at 5–10 °C a solution of phthalylglycyl chloride **4** (14.64 g,

65,48 mmol) in $CHCl_3$ (50 mL) was added dropwise over 30 min. During the chloride addition the product precipitated. The mixture was stirred additionally at 24 ^oC for 30 min and then chloroform (1.50 L) was added to completely dissolve the product. The organic phase was washed with water (300 mL), 5% sodium carbonate solution (300 mL) and water (300 mL) and dried over $MgSO_4$. The solution was concentrated in vacuo to 1/3 of the initial volume and after cooling to 10 °C the precipitated product was filtered off to give 17.60 g (87%) of product **5** as pale beige solid; mp = 234-235 °C. IR (KBr): 3269, 3063, 3026, 1772, 1732, 1666, 1587, 1541, 1413, 1257, 1211, 952, 715 cm^{-1}; ^1H NMR (500 MHz, $CDCl_3$): δ 3.99 (s, 2 H), 4.30 (s, 2 H), 7.05-7.18 (m, 7 H, Ar), 7.22-7.30 (m, 2H, Ar), 7.75-7.77 (m, 2 H, Ar), 7.85-7.87 (m, 2 H, Ar); ^{13}C NMR (125 MHz, $CDCl_3$): δ 38.3, 41.6, 123.8, 124.1, 125.8, 126.7, 127.7, 128.1, 128.9, 128.9, 131.0, 131.4, 131.9, 134.3, 135.1, 138.7, 164.2, 167.6; HRMS (ESI): *m/z* calcd for $C_{23}H_{18}N_2O_3Na$ $[M+Na]^+$: 393.1215; found: 393.1223.

2-(11*H*-Dibenzo[*b*,*e*]azepin-6-ylmethyl)isoindole-1,3-dione (6)

The suspension of **5** (4.0 g, 10.80 mmol) in dry CH_3CN (40 mL) and $POCl_3$ (40 mL, 214.57 mmol) were heated at reflux for 3 h. The reaction mixture was concentrated in vacuo and 150 mL of $CHCl_3$ was added. After cooling to 5 °C the organic solution was alkalized with 50 mL of a 10% solution of NH_3 (aq). After phase separation, the water layer was extracted with $CHCl_3$ (80 mL). The combined organic layers were washed with water (50 mL), dried over Na_2SO_4 and concentrated under reduced pressure. The isolation by column chromatography on a silica gel with $CHCl_3$ gave compound **6** (1.38 g, 36%) as pale yellow crystals; mp = 208-209 ^oC ([2] mp = 205.5-207.5 °C). IR (CCl_4): 1776, 1724, 1419, 1392, 1107, 949 cm^{-1}; ^1H NMR (500 MHz, $CDCl_3$): δ 3.61 (s, 2 H), 5.09 (s, 2 H), 7.04-7.16 (m, 4 H, Ar), 7.25-7.28 (m, 2 H, Ar), 7.36-7.39 (m, 1 H, Ar), 7.57-7.59 (m, 1 H, Ar), 7.68-7.72 (m, 2 H, Pht), 7.84-7.88 (m, 2 H, Pht); ^{13}C NMR (125 MHz, $CDCl_3$): δ 38.7, 44.6, 123.4, 125.3, 126.2, 126.2,

126.5, 126.8, 127.0, 127.0, 130.6, 131.3, 132.3, 133.9, 143.3, 144.8, 161.8, 168.2. HRMS (ESI): *m/z* calcd for $C_{23}H_{17}N_2O_2$ $[M+H]^+$: 353.1290; found: 353.1281.

(6*S*)-2-(6,11-Dihydro-5*H*-dibenzo[*b*,*e*]azepin-6-ylmethyl)isoindole-1,3-dione (7)

The catalyst (*R*,*R*)-**11** was preformed from $[RuCl_2(C_6H_6)]_2$ (9.25 mg, 18.5 µmol), *N*-tosyl-(1*R*,2*R*)-cyclohexanediamine (TsCYDN) (11.41 mg, 42.53 µmol) and Et_3N (51.04 µmol) in 6 mL CH_2Cl_2. To a solution of imine **6** (0.65 g, 1.85 mmol) in CH_2Cl_2 (25 mL) a 5:2 formic acid/triethylamine mixture (5.0 mL) was introduced followed by the addition of the pre-formed catalyst. The mixture was then stirred at 23–25 °C for 24 h. After this time CH_2Cl_2 (20 mL) and water (10 mL) were added and the reaction mixture was alkalized with solid K_2CO_3. The layers were separated and the water layer was extracted twice with CH_2Cl_2 (2 × 20 mL). The combined organic layers were washed with brine (20 mL), dried over $MgSO_4$ and the solvent was evaporated in vacuo. The residual oil was purified by column chromatography on silica gel using CH_2Cl_2 as eluent to afford 436 mg (65%) of compound (*S*)-(+)-**5** as pale yellow crystals $[\alpha]_D^{23}$ = +62.8 (*c* 1; $CHCl_3$); recrystallization from chloroform: methanol: diethyl ether (24 mL:24 mL:12 mL) mixture gave 400 mg of pure enantiomer; $[\alpha]_D^{23}$ = +68.1 (*c* 1, $CHCl_3$); mp = 244.7–245.5 °C. IR (KBr): 3373, 3034, 2939, 1774, 1697, 1604, 1496, 1390, 1274, 1074, 962, 758, 713 cm^{-1}; ^1H NMR (500 MHz, $CDCl_3$): δ 3.88 (d, *J* = 15.0 Hz, 1 H, methylene bridge), 4.24-4.34 (m, 2 H), 4.46 (d, *J* = 15.0 Hz, 1 H, methylene bridge), 4.22 (dd, J_1 = 5.0 Hz, J_2 = 9.0 Hz, 1 H, methine), 6.47 (d, *J* = 8.0 Hz, 1 H, Ar), 6.60-6.63 (m, 1 H, Ar), 6.90-6.93 (m, 1 H, Ar), 7.00 (d, *J* = 6.5 Hz, 1 H, Ar), 7.15-7.25 (m, 4 H, Ar), 7.71-7.74 (m, 2 H, Pht), 7.84-7.88 (m, 2 H, Pht); ^{13}C NMR (125 MHz, $CDCl_3$): δ 39.8, 42.3, 55.0, 117.9, 118.7, 123.5, 124.0, 124.7, 127.0, 127.5, 128.2, 130.1, 131.9, 134.2, 136.8, 139.6, 144.8, 168.7; HRMS (ESI): *m/z* calcd for $C_{23}H_{18}N_2O_2Na$ $[M+Na]^+$: 377.1266; found: 377.1275.

Monocrystals of **7** suitable for crystallographic measurements were obtained from a chloroform/methanol/diethyl ether mixture by slow evaporation. The absolute structure of the studied crystal, and hence the absolute configuration of the compound was determined based on the value of the Flack parameter [3]. Since its value for the structure shown in Figure 1 was approximately 0, the molecular structure has the depicted configuration. The data were deposited with Cambridge Crystallographic Data Centre under the number CCDC 1058969.

(6*R*)-2-(6,11-Dihydro-5*H*-dibenzo[*b*,*e*]azepin-6-ylmethyl)isoindole-1,3-dione (7)

The enantiomer (6*R*)-(−)-**5** was obtained as a pale yellow crystals using (*S*,*S*)-**11** catalyst formed from $[RuCl_2(C_6H_6)]_2$ and *N*-tosyl-(1*S*,2*S*)-cyclohexanediamine (TsCYDN); $[\alpha]_D^{23}$ = -62.2; recrystallization from chloroform/methanol/diethyl ether (1:1:0.5) mixture gave pure enantiomer; $[\alpha]_D^{23}$ = −68.3 (*c* 1, $CHCl_3$); mp = 244.1–245.1 °C.

(±)-2-(6,11-Dihydro-5*H*-dibenzo[*b*,*e*]azepin-6-ylmethyl)isoindole-1,3-dione (7) [4]

To a stirred solution of imine **6** (0.22 g, 0.62 mmol) in a mixture of DMF (120 mL) and formic acid (57.4 mg, 1.25 mmol) 10% Pd/C (50 mg) was added and the resulting suspension was shaken for 3 hours at 70 °C in a hydrogen atmosphere (balloon). The ethanolic solution was filtered through celite and the solvent was evaporated *in vacuo*. The oily residue was purified with column chromatography on silica gel using CH_2Cl_2 as eluent to afford 0.21 g (95%) of racemic amine **7** as pale yellow solid; mp = 214.2–215.6 °C.

(6*S*)-(−)-[6-(1,3-Dioxo-1,3-dihydroisoindol-2-ylmethyl)-6,11-dihydro-dibenzo[*b*,*e*]azepin-5-yl]oxoacetic acid ethyl ester (8)

To a stirred suspension of (*S*)-(+)- **7** (256 mg, 0.72 mmol) and triethylamine (0.15 mL, 1.08 mmol) in 10 mL of dry CH_2Cl_2 at 25 °C the solution of ethyl oxalyl chloride (0.12 mL, 1.08 mmol) in 4 mL of CH_2Cl_2 was added over 10 min. During addition of chloride

a clear solution was formed. After 15 min CH_2Cl_2 (10 mL), water (6 mL) and 10% HCl_{aq} solution (1.5 mL) were added. The layers were separated and the organic layer was washed with water (2 × 5 mL) and dried over Na_2SO_4. After evaporation of the solvent, the residual oil was purified with column chromatography on silica gel using chloroform as eluent. The purification gave 308 mg (94%) of compound (*S*)-(−)-**8** as colorless crystals; $[\alpha]_D^{23}$ = −307 (*c* 1, $CHCl_3$); mp = 172–173 °C. IR (CCl_4): 1720, 1680, 1498, 1394, 1354, 1211, 1016 cm^{-1}; ^1H NMR (500 MHz, $CDCl_3$): δ 0.94 (t, *J* = 7.5 Hz, 3 H, CH_3), 3.63 (d, *J* = 15.0 Hz, 1 H, methylene bridge), 3.81 (dd, J_1 = 2.0 Hz, J_2 = 14.5 Hz,1 H), 3.90-4.01 (m, 2 H), 4.65 (dd, J_1 = 4.0 Hz, J_2 = 14.5 Hz,1 H), 4.67 (d, *J* = 15.0 Hz, 1 H, methylene bridge), 6.59 (d, *J* = 9.0 Hz, 1 H, methine), 7.18-7.29 (m, 3 H, Ar), 7.35-7.40 (m, 3 H, Ar), 7.71-7.80 (m, 4 H, Ar), 7.88-7.93 (m, 2 H, Ar); ^{13}C NMR (125 MHz, $CDCl_3$): δ 13.5, 39.5, 44.2, 55.0, 61.6, 123.5, 127.2, 127.6, 127.6, 128.9, 129.8, 130.0, 130.4, 132.0, 134.2, 135.0, 135.4, 140.8, 162.0, 162.3, 168.4; HRMS (ESI): *m/z* calcd for $C_{27}H_{23}N_2O_5$ $[M+H]^+$: 455.1607; found: 455.1620.

(±)-(-)-[6-(1,3-Dioxo-1,3-dihydroisoindol-2-ylmethyl)-6,11-dihydro-dibenzo[*b*,*e*]azepin-5-yl]oxoacetic acid ethyl ester (8)

The racemate was prepared in the same manner as pure enantiomer; mp = 210.5–211.2 °C.

(*S*)-(+)-1,2,9,13b-Tetrahydro-2,4a-diazatribenzo[*a*,*c*,*e*]cycloheptene-3,4-dione (9)

Compound **8** (0.29 g, 0.64 mmol) and hydrazine (0.12 mL, 3.83 mmol) in 12 mL of 99.8% ethanol were heated at reflux for 2 h. After evaporation of volatile components, the residual solid was purified by column chromatography on silica gel using chloroform/methanol 0–2% MeOH as a solvent system to afford 153 mg (90% yield) of compound (*S*)-(+)-**9** as colorless solid; $[\alpha]_D^{23}$ = +274.6 (*c* 1, $CHCl_3$); mp = 236–237 °C. IR (KBr): 3186, 3030, 2885, 1722, 1683, 1489, 1417, 1346, 1070, 754 cm^{-1}; ^1H NMR (500 MHz, $CDCl_3$): δ 3.46 (d, *J* = 13.5 Hz, 1 H, methylene bridge) 3.58 (dt, J_1 =

4.0 Hz, J_2 = 13.5 Hz, 1 H), 3.90 (t, J = 13.0 Hz, 1 H), 4.49 (d, J = 13.5 Hz, 1 H, methylene bridge), 5.27 (dd, J_1 = 3.5 Hz, J_2 = 11.0 Hz,1 H, methine), 7.05-7.07 (m, 1 H, Ar), 7.12-7.20 (m, 4 H, Ar), 7.22-7.26 (m, 2 H, Ar), 7.35 (d, J = 8.0 Hz, 1 H, Ar), 8.87 (d, J = 3.0 Hz, 1 H, NH); ^{13}C NMR (125 MHz, $CDCl_3$): δ 38.3, 46.2, 61.5, 127.4, 127.5, 127.7, 128.3, 128.4, 128.6, 128.9, 129.3, 132.9, 136.3, 139.2, 140.2, 158.0, 158.9. HRMS (ESI): *m/z* calcd for $C_{17}H_{14}N_2O_2Na$ $[M+Na]^+$: 301.0953; found: 301.0945.

(±)-(+)-1,2,9,13b-Tetrahydro-2,4a-diazatribenzo[*a*,*c*,*e*]cycloheptene-3,4-dione (9)

The racemate was prepared in the same manner as pure enantiomer; mp = 274–275 °C.

(14b*S*)-(+)-1,2,3,4,10,14b-Hexahydrodibenzo[*c*,*f*]pyrazino[1,2-*a*]azepine (10)

To a stirred suspension of **9** (100 mg, 0.36 mmol) in 10 mL of dry diethyl ether was added 3.0 mL of 1.0 M $LiAlH_4$ solution in THF, and then the suspension was stirred at 25 °C for 1 h and heated at reflux for 0.5 h. After cooling to room temperature, 30 mL of Et_2O and 0.6 mL of water were added to the reaction mixture, the precipitate was filtered off and additionally extracted with Et_2O (5 × 30 mL). The combined organic layers were dried over $MgSO_4$. After evaporation of the solvent, the residual oil was purified by column chromatography on alumina using chloroform/methanol 0–2% MeOH as a solvent system to afford 72 mg (81% yield) of compound (*S*-(+)-**10** in the form of beige solid; $[\alpha]_D^{23}$ = +502 (*c* 0. 5, MeOH) {[5] $[\alpha]_D^{25}$ = +504 (*c* 1, DMF; [6] $[\alpha]_D^{23}$ = −497 (*c* 1, MeOH) for (*R*)-isomer}; mp = 112–113 °C ([5] mp = 128–130 °C). IR (CCl_4): 3064, 3022, 2926, 2854, 1681, 1492, 1446, 1263, 1236, 1134 cm^{-1}; ^{1}H NMR (500 MHz, $CDCl_3$): δ 1.86 (bs, 1 H, NH), 3.00-3.15 (m, 4 H), 3.20-3.27 (m, 2 H), 3.29 (d, J = 12.5 Hz, 1 H, methylene bridge), 3.94 (dd, J_1 = 2.5 Hz, J_2 = 10.0 Hz, 1 H, methine), 4.85 (d, J = 13.0 Hz, 1 H, methylene bridge), 6.86 (td, J_1 = 1.5 Hz, J_2 = 7.5 Hz, 1 H, Ar), 6.95-7.13 (m, 6 H, Ar), 7.17 (m, 1 H, Ar); ^{13}C NMR (125 MHz, $CDCl_3$): δ

38.8, 46.6, 52.8, 56.0, 68.8, 118.9, 122.2, 126.5, 126.5, 126.9, 127.2, 128.1, 129.4, 137.4, 139.2, 140.0, 148.9. HRMS (ESI): *m/z* calcd for $C_{17}H_{19}N_2$ $[M+H]^+$: 251.1548; found: 251.1559.

(±)-1,2,3,4,10,14b-Hexahydrodibenzo[*c*,*f*]pyrazino[1,2-*a*]azepine (10)

The racemate was prepared in the same manner as pure enantiomer; pale brown oil ([7] oil; [8] mp = 83–84 °C).

(14b*S*)-(+)-1,2,3,4,10,14b-Hexahydro-2-methyldibenzo[*c*,*f*]pyrazino[1,2-*a*]azepine (1)

To a stirred solution of **10** (42 mg, 0.17 mmol) in 8 mL of dry THF potassium carbonate (24.35 mg, 0.18 mmol) was added and then the solution of methyl iodide (10.4 µL, 0.17 mmol) in 1 mL THF was introduced. The suspension was stirred at 24-25 °C for 2.5 h and evaporated in vacuo. To the residue 26 mL of $CHCl_3$ and 8 mL of water was added. The layers were separated and the water layer was extracted twice with 8 mL $CHCl_3$. The combined organic layers were dried over $MgSO_4$ and after evaporation of the solvent, the residual brown oil was purified with column chromatography on silica gel using chloroform/methanol 0–1% MeOH as a solvent system to afford 30 mg (67% yield) of compound (*S*)-(+)-**1** in the form of solidifying oil; during purification step a small degree of product decomposition was observed; $[\alpha]_D^{23}$ = +469.2 (*c* 1, $CHCl_3$); $[\alpha]_D^{23}$ = +436.5 (*c* 1, EtOH) {[9] $[\alpha]_D^{23}$ = +450 (*c* 0.26, EtOH)}; $[\alpha]_D^{23}$ = +428.0 (*c* 0.5, MeOH) {[5] $[\alpha]_D^{25}$ = +469.0 (*c* 1, MeOH)}. Enantiomeric purity was determined by HPLC analysis (Chiracel OD-H, hexane:2-propanol = 80:20, 1ml/min, *S* isomer 5.6min). IR (CCl_4): 3064, 3022, 2939, 2794, 1492, 1446, 1251, 1132 cm^{-1}; ^1H NMR (500 MHz, $CDCl_3$): δ 2.37-2.42 (m, 4 H), 2.46 (t, J = 10.5 Hz, 1 H), 2.92 (dt, J_1 = 2.0 Hz, J_2 = 11.0 Hz, 1 H), 3.02 (dd, J_1 = 1.5 Hz, J_2 = 11.0 Hz, 1 H), 3.25-3.28 (m, 1 H), 3.30 (d, J = 13.0 Hz, 1 H, methylene bridge), 3.42 (td, J_1 = 3.0 Hz, J_2 = 11.0 Hz, 1 H), 4.14 (dd, J_1 = 2.0 Hz, J_2 = 10.0 Hz, 1 H,

methine), 4.81 (d, J = 13.0 Hz, 1 H, methylene bridge), 6.87 (td, J_1 = 1.0 Hz, J_2 = 7.5 Hz, 1 H, Ar), 7.00-7.02 (m, 2 H, Ar), 7.05-7.13 (m, 4 H, Ar), 7.16 (td, J_1 = 1.5 Hz, J_2 = 7.5 Hz, 1 H, Ar); ^{13}C NMR (125 MHz, $CDCl_3$): δ 38.8, 45.6, 51.0, 55.4, 64.6, 66.2, 119.0, 122.3, 126.5, 126.6, 127.0, 127.3, 128.1, 129.5, 137.1, 139.3, 139.8, 148.4. HRMS (ESI): *m/z* calcd for $C_{18}H_{21}N_2$ $[M+H]^+$: 265.1705; found: 265.1712.

(±)-1,2,3,4,10,14b-Hexahydro-2-methyldibenzo[*c*,*f*]pyrazino[1,2-*a*]azepine (1)

The racemate was prepared in the same manner as pure enantiomer; mp = 109.5-110.5 °C ([10] mp = 111–113 °C). The HPLC analysis (Chiracel OD-H, hexane/2-propanol = 80:20, 1mL/min, *R* isomer 5.0 min and *S* isomer 5.6 min).

(6*S*)-(+)-6-Aminomethyl-6,11-dihydro-5*H*-dibenzo[*b*,*e*]azepine (13)

Compound **7** (100 mg, 0.28 mmol) and hydrazine (0.35 mL, 11.16 mmol) in 4 mL of 99.8% ethanol were heated at reflux for 5 h. After evaporation of volatile components, the residual solid was purified with column chromatography on silica gel using chloroform/methanol 0–3% MeOH as a solvent system to afford 57 mg (90% yield) of compound (*S*)-(+)-**13** as pale beige solid; $[\alpha]_D^{23}$ = +160.1 (*c* 1, $CHCl_3$); mp = 90–91.5 °C. IR (CCl_4): 3356, 3061, 3022, 2947, 2868, 1604, 1477, 1315, 1257, 114 cm^{-1}; ^{1}H NMR (300 MHz, $CDCl_3$): δ 1.39 (bs, 2H, NH_2), 3.07-3.28 (m, 2 H), 3.92 (d, J = 25.0 Hz, 1 H, methylene bridge), 4.32 (d, J = 25.0 Hz, 1 H, methylene bridge), 4.70 (m, 1 H, methine), 6.62 (dd, J_1 = 2.0 Hz, J_2 = 13.0 Hz, 1 H, Ar), 6.68 (td, J_1 = 2.0 Hz, J_2 = 12.5 Hz, 1 H, Ar), 6.96-7.08 (m, 3 H, Ar), 7.16-7.21 (m, 3 H, Ar); ^{13}C NMR (75 MHz, $CDCl_3$): δ 39.9, 46.9, 59.0, 118.6, 119.3, 125.8, 126.6, 126.8, 127.4, 127.5, 128.6, 129.7, 137.98, 139.6, 145.8; HRMS (ESI): *m/z* calcd for $C_{15}H_{17}N_2$ $[M+H]^+$: 225.1392; found: 225.1384.

(13b*S*)-(+)-3-Amino-9,13b-dihydro-1*H*-dibenz[*c*,*f*]imidazo[1,5-*a*]azepine (2)

To a stirred at 25 °C solution of amine **13** (57 mg, 0.25 mmol) in 1 mL of 99.8% ethanol, solution of BrCN (30 mg, 0.28 mmol) in 0.5 mL of THF was added. After 2 h

to the reaction mixture ethanol (6 mL) and 25% NH_4OH solution (44.40 μL, 0.56 mmol) were added and the mixture was concentrated. The residual oil was purified with column chromatography on silica gel using chloroform/methanol 0–2% MeOH as a solvent system to afford 61 mg (96% yield) of compound (*S*)-(+)-**2** as pale beige solid; $[\alpha]_D^{23}$ = +220.6 (*c* 1, $CHCl_3$); mp = 279–280 °C. IR (KBr): 3203, 3099, 1662, 1585, 1558, 1489, 758 cm^{-1}; ^1H NMR (500 MHz, $CDCl_3$): δ 2.02 (bs, NH-tautomeric form); 3.58 (d, *J* = 14.5 Hz, 1 H, methylene bridge), 3.72 (t, *J* = 9.5 Hz, 1 H, CH_2N), 4.39 (t, *J* = 9.5 Hz, 1 H, CH_2N), 4.52 (d, *J* = 14.5 Hz, 1 H, methylene bridge), 5.43 (t, *J* = 10.0 Hz,1 H, methine), 7.01-7.03 (m, 1 H, Ar), 7.21-7.28 (m, 3 H, Ar), 7.36-7.49 (m, 4 H, Ar), 8.85 (bs, 1H, NH_2); ^{13}C NMR (125 MHz, $CDCl_3$): δ 38.4, 50.7, 63.0, 126.3, 127.8, 128.0, 128.4, 129.0, 129.5, 130.2, 130.7, 132.1, 134.2, 134.7, 158.1. HRMS (ESI): *m/z* calcd for $C_{16}H_{16}N_3$ $[M+H]^+$: 250.1344; found: 250.1353.

References

1. Sheldrick, G. M. *Acta Crystallogr., Sect. A* **2008**, 64, 112-122.
2. Moffett, R. B. *J. Heterocyclic Chem.* **1980**, 17, 341-350.
3. Flack, H. D. *Acta Crystallogr., Sect. A* **1983**, 39, 876-881.
4. Schneider, H. Verfahren zur Herstellung von 3-Amino-9,13b-dihydro-1*H*-dibenz[*c*,*f*]imidazol[1,5-*a*]azepin-hydrochlorid. E.P. Patent 0 496 306 A1, January 18, 1992.
5. Fukumi, H.; Sakamoto, T.; Sugiyama, M.; Lizuka, Y.; Yamaguchi, T. Tetracyclic compounds having anti-allergic and anti-asthmatic activities and their use. U.S. Patent 5,362,725, November 8, 1994.
6. Hiroshi, T.; Hisayoshi, Y.; Katsuyuki, O. *Bull. Chem. Soc. Jpn*, **1996**, 69, 3581-3590.

7. Filer, C. N.; Fazio, R.; Ahern, D. G. *J. Org. Chem.*, **1981**, 46, 3344-3346.

8. Copp, F. C.; Boura, A. L. A.; Jackson, W. R.; Cullen, J. D. 1,2,3,4,10,14b-hexahydrodibenzo[c,f]pyrazino-[1,2-a]azepino derivatives and 10-aza, 10-oxa and 10-thia analogues. U.S. Patent 5,049,637, September 17, 1991.

9. Cirilli, R.; Orlando, V.; Ferretti, R.; Turchetto, L.; Silvestri, R.; De Martino, G.; La Torre, F. *Chirality*, **2006**, 18, 621-632.

10. Hulinska, H.; Polivka, Z.; Jilek, J.; Šindelař, K.; Holubek, J.; Svatek, E.; Matoušowa, O.; Buděšinsky, M.; Frycova, H.; Protiva, M. *Coll. Czech. Chem. Commun.*, **1988**, 53, 1820-1844.

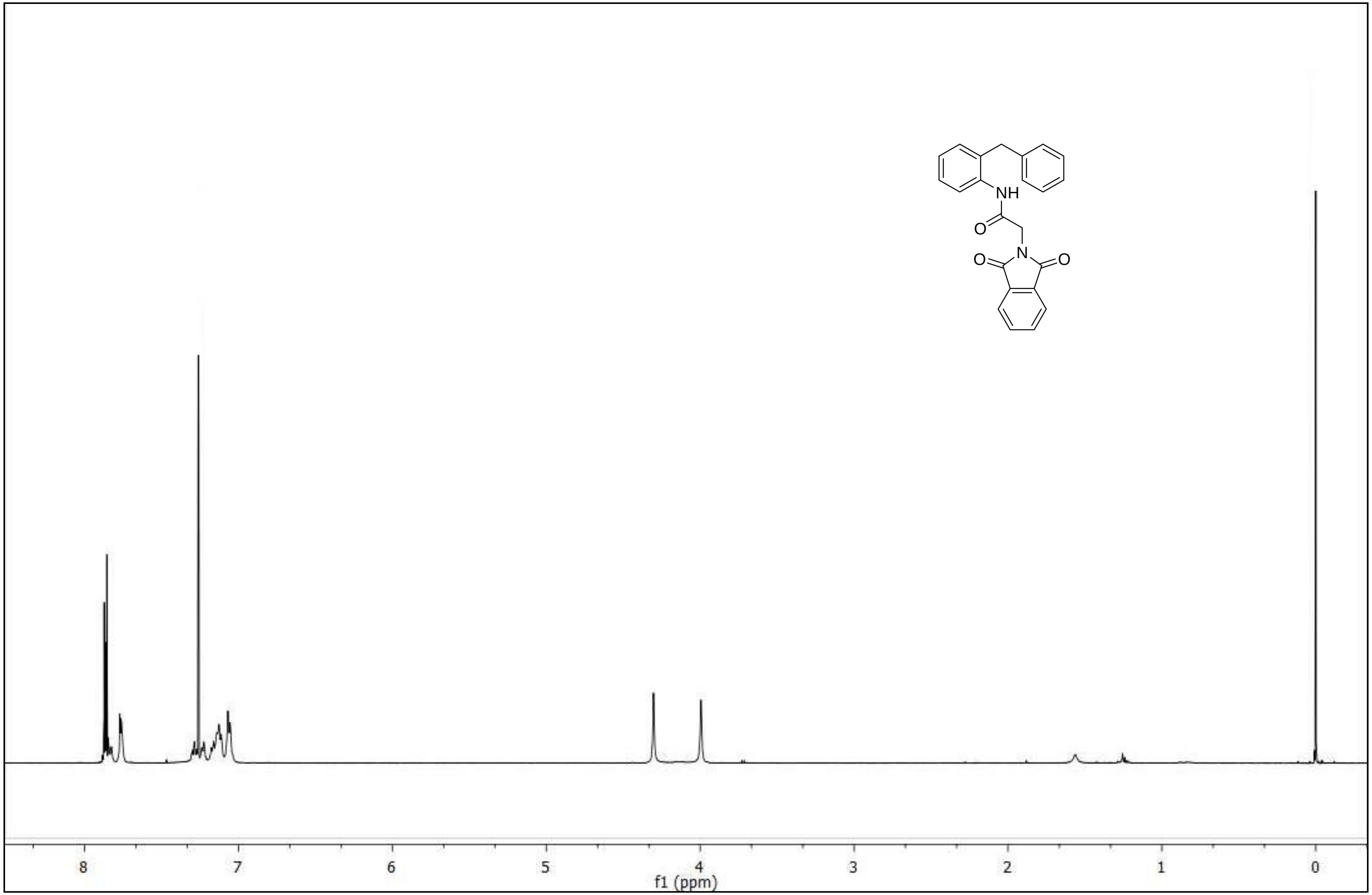

NH
O
N
O
O
8
7
6
5
4
3
2
1
0
f1 (ppm)

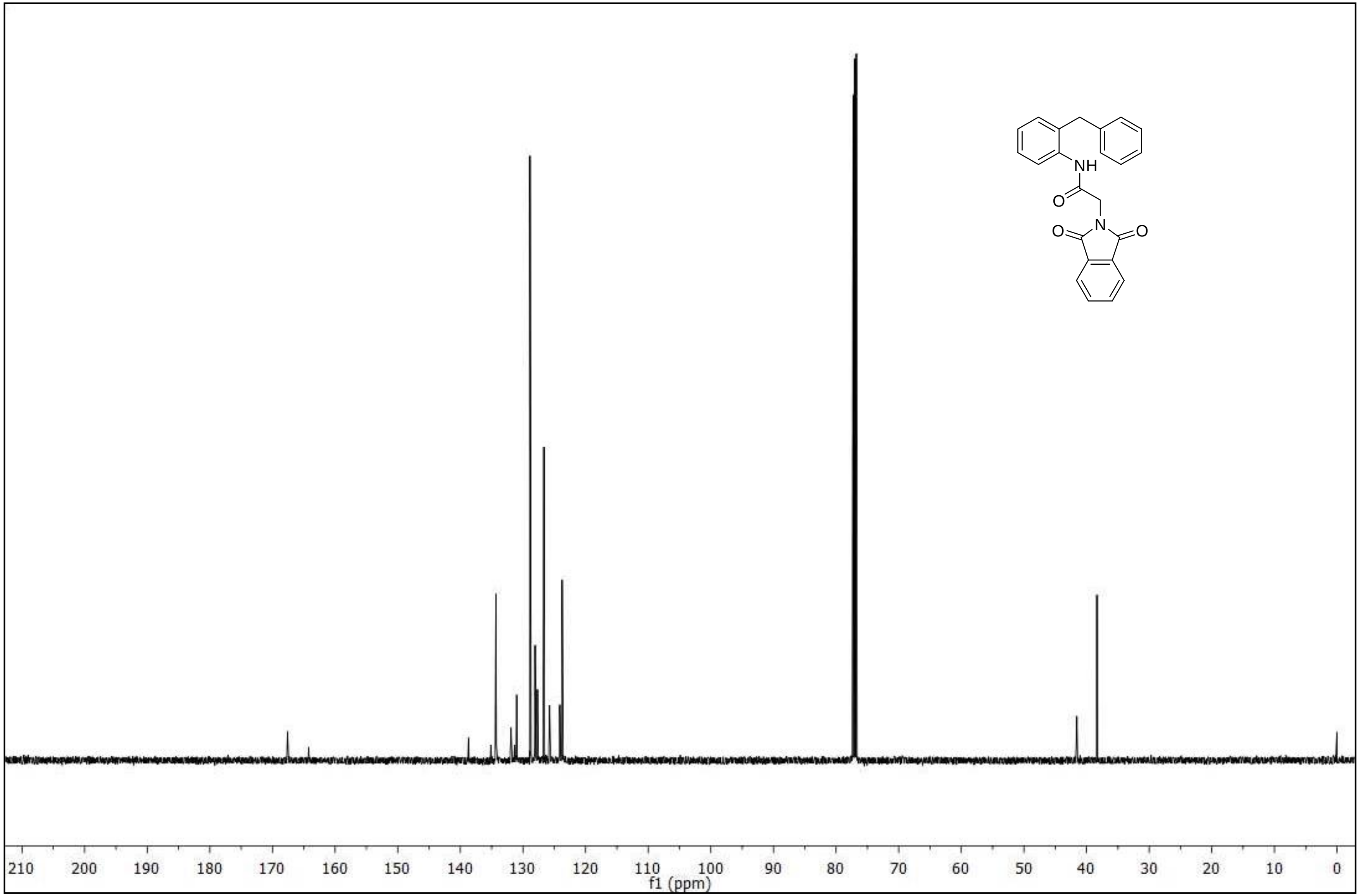

NH
O
N
O
O
210
200
190
180
170
160
150
140
130
120
110
100
90
80
70
60
50
40
30
20
10
0
f1 (ppm)

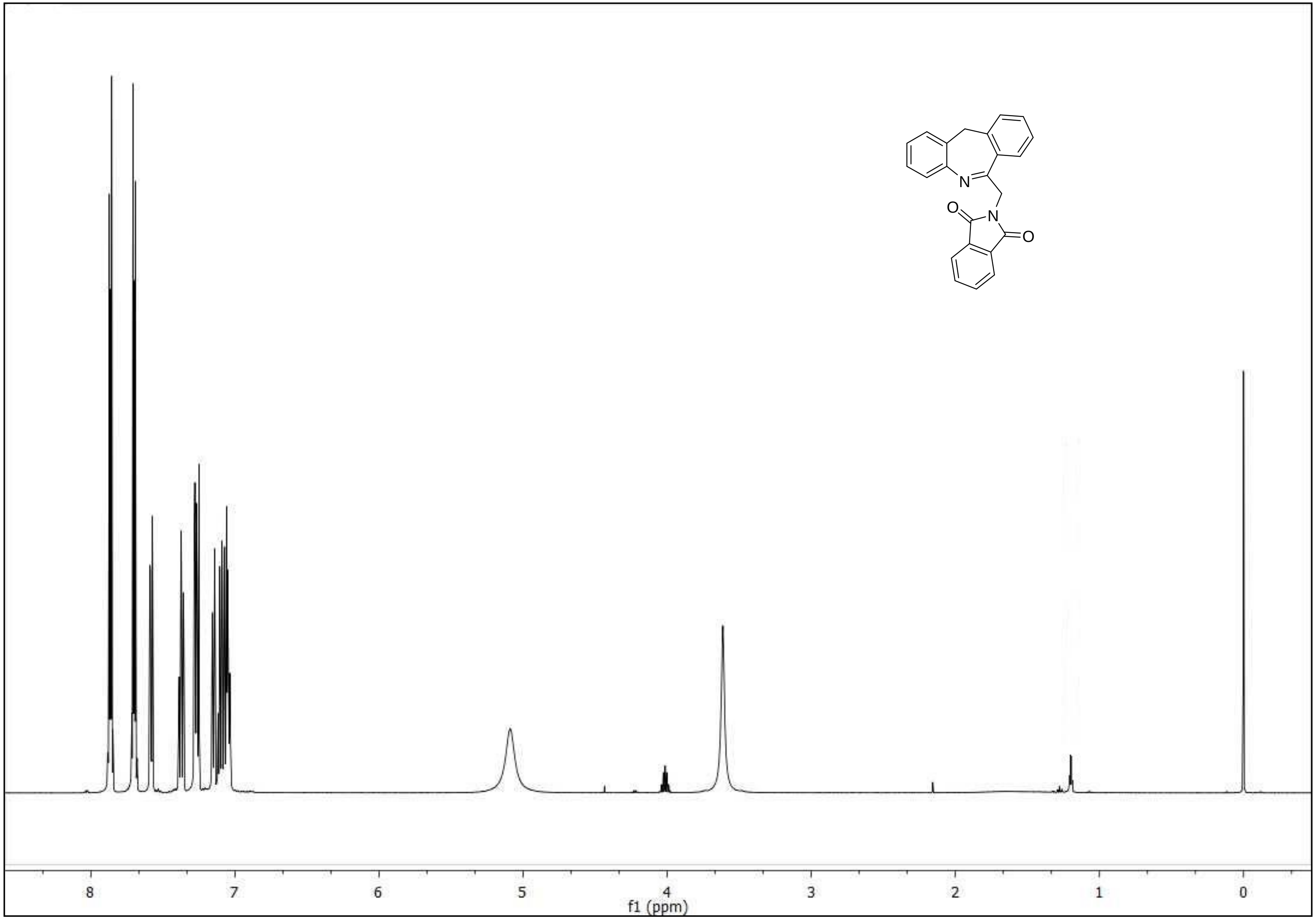
N
O
N
O
8
7
6
5
4
3
2
1
0
f1 (ppm)

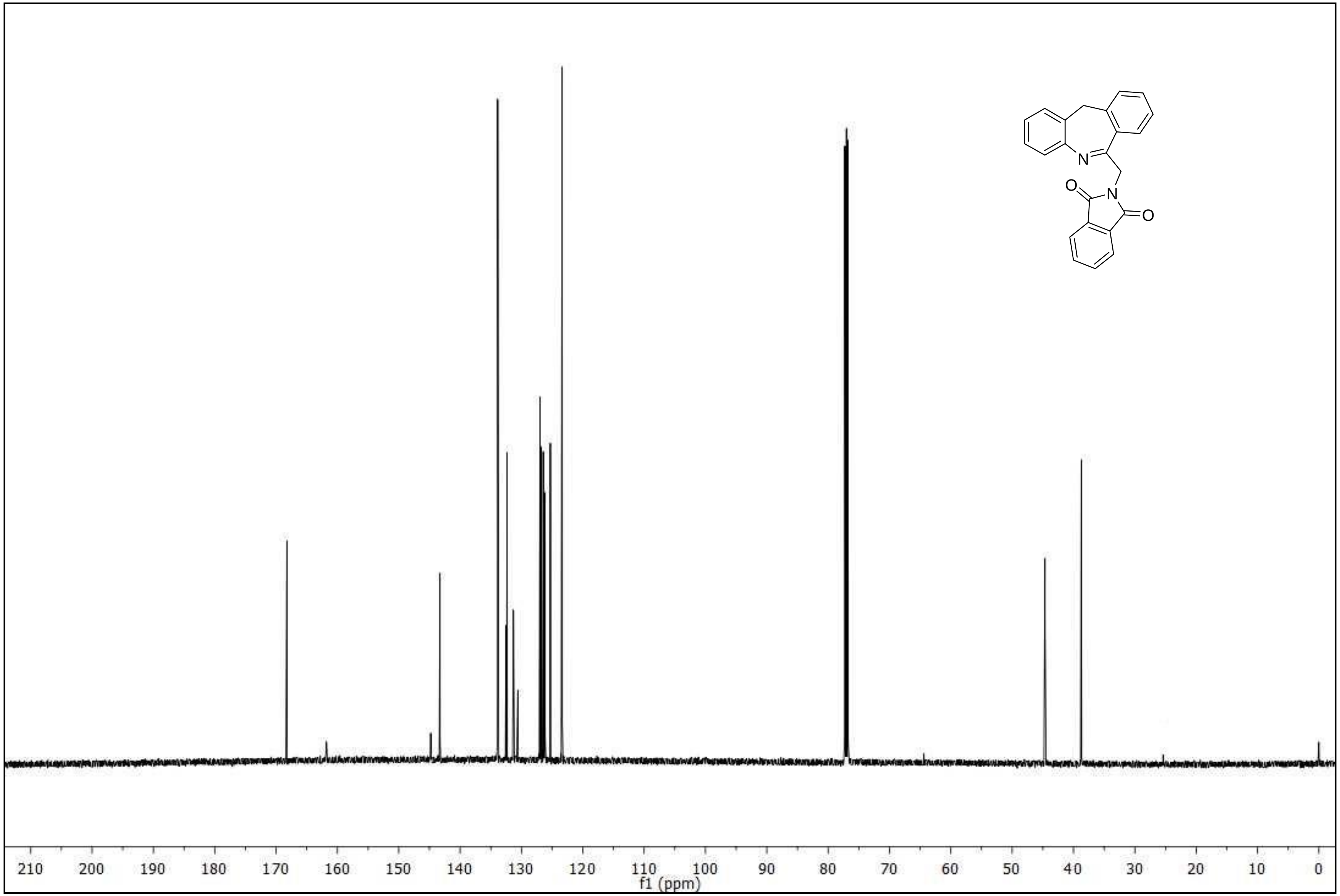
N
O
N
O
210
200
190
180
170
160
150
140
130
120
110
100
90
80
70
60
50
40
30
20
10
0
f1 (ppm)

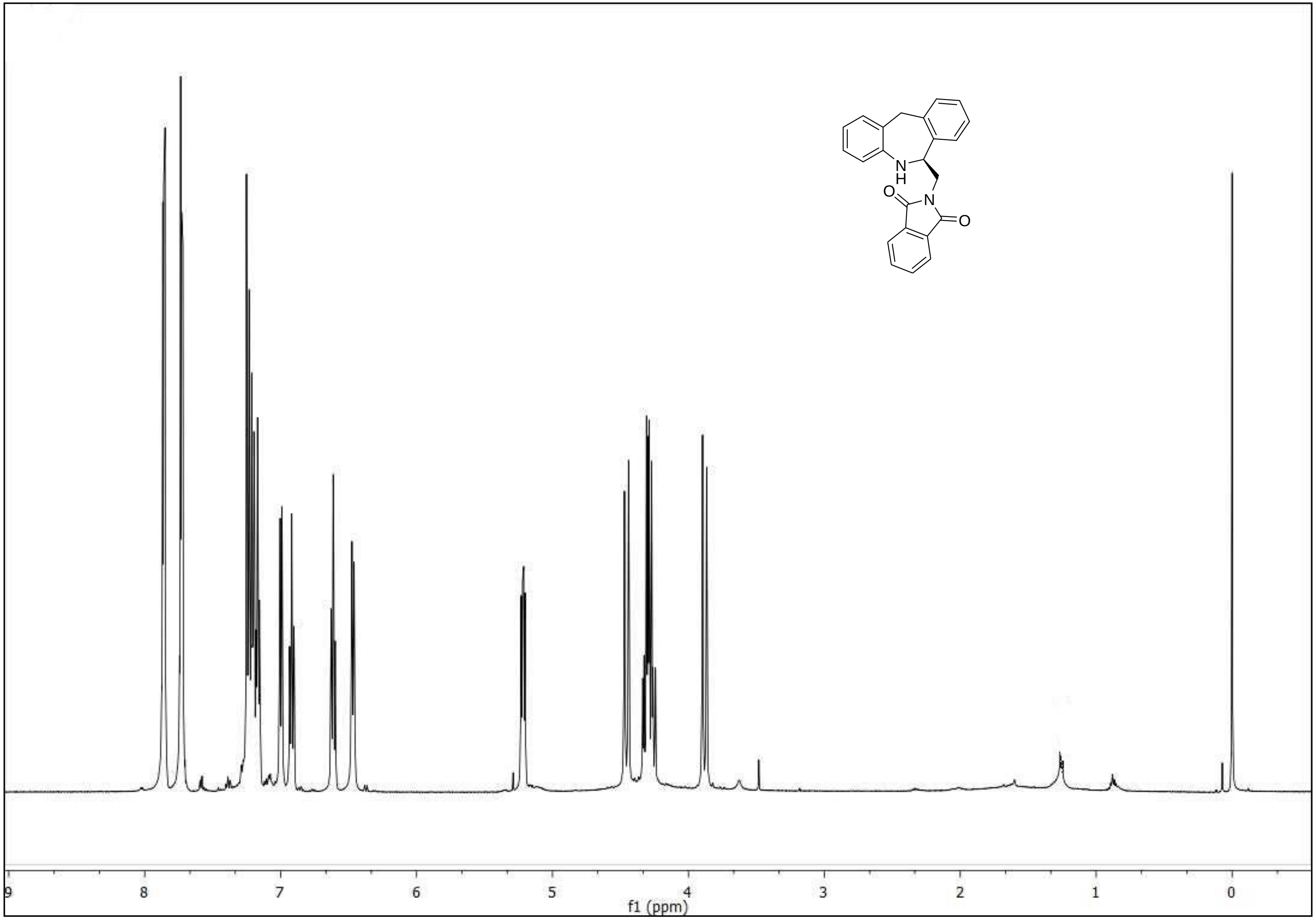

O
N
O
N
H
f1 (ppm)
9
8
7
6
5
4
3
2
1
0

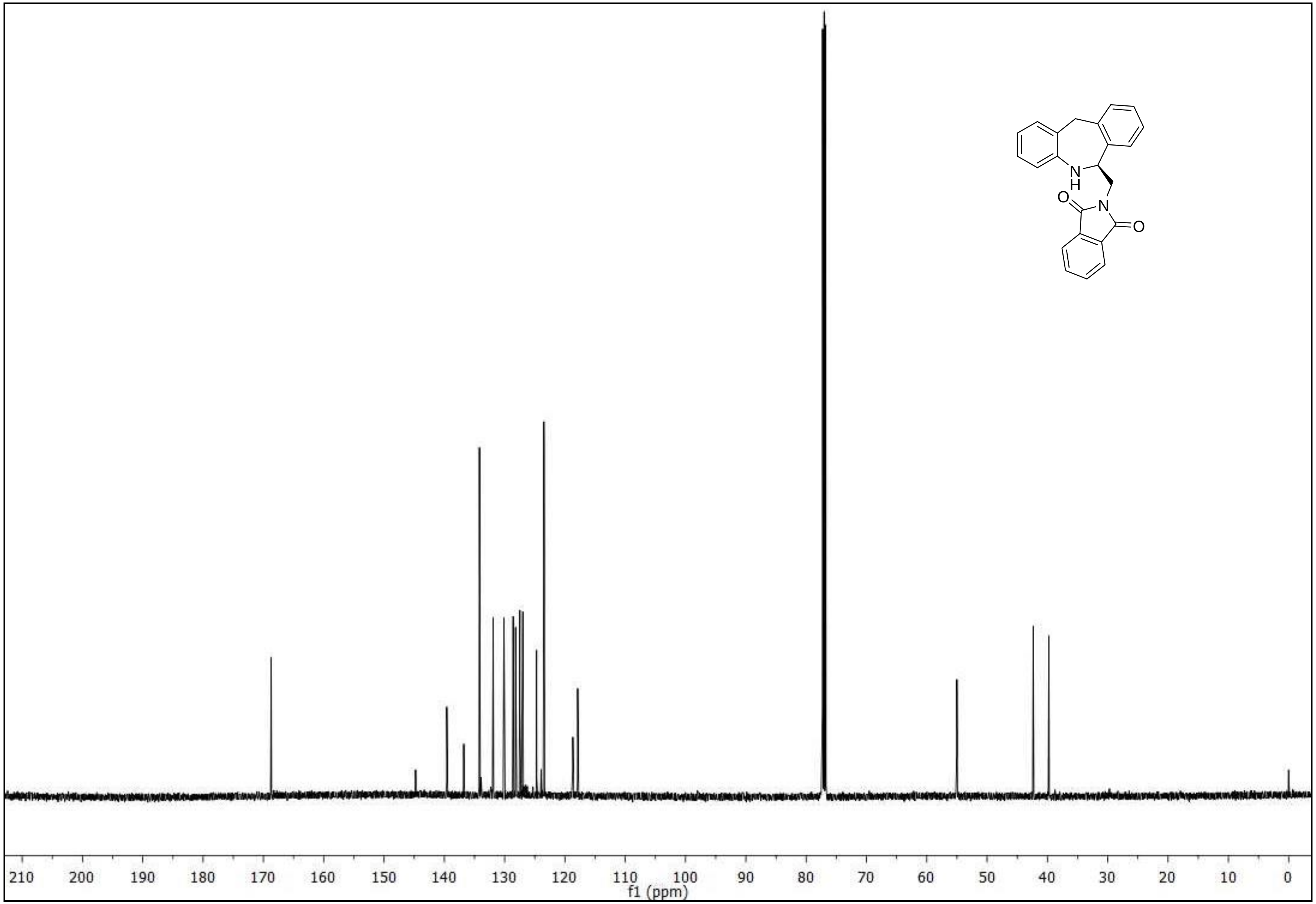

H
N
O
N
O
210
200
190
180
170
160
150
140
130
120
110
100
90
80
70
60
50
40
30
20
10
0
f1 (ppm)

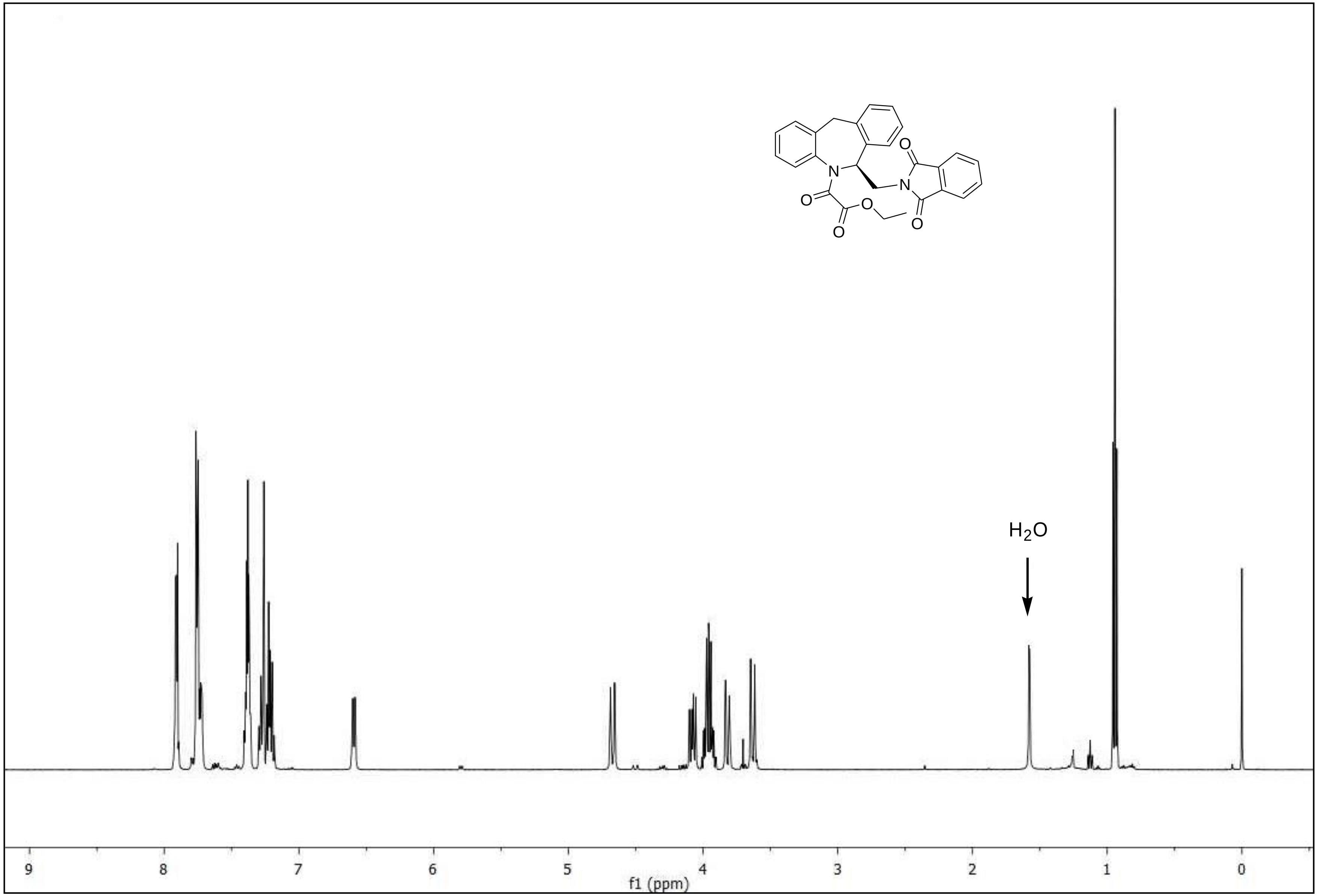

N
O
O
O
N
O
O
H2O
9
8
7
6
5
4
3
2
1
0
f1 (ppm)

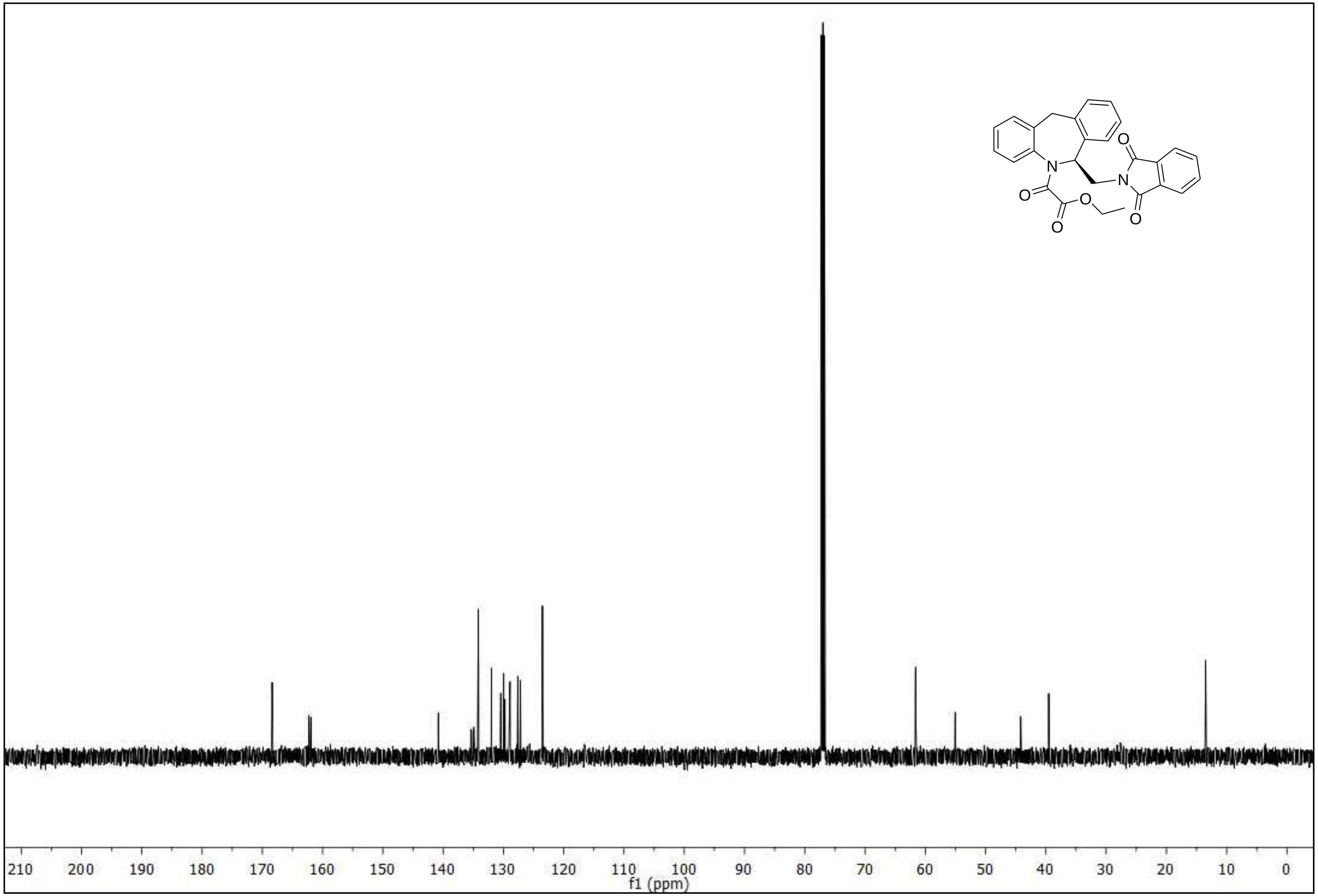
210 200 190 180 170 160 150 140 130 120 110 100 90 80 70 60 50 40 30 20 10 0
f1 (ppm)

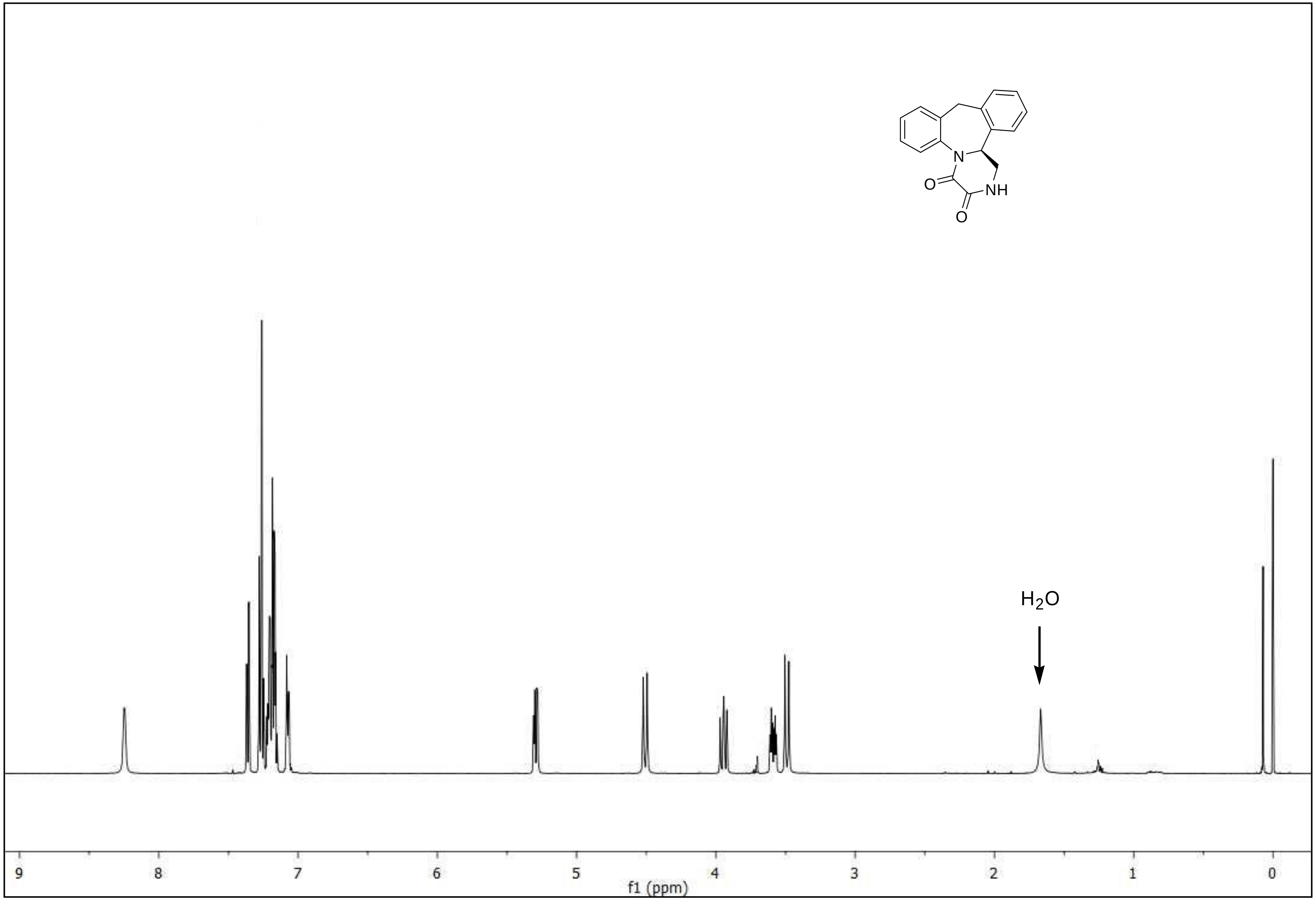
O
NH
O
N
H2O
9
8
7
6
5
4
3
2
1
0
f1 (ppm)

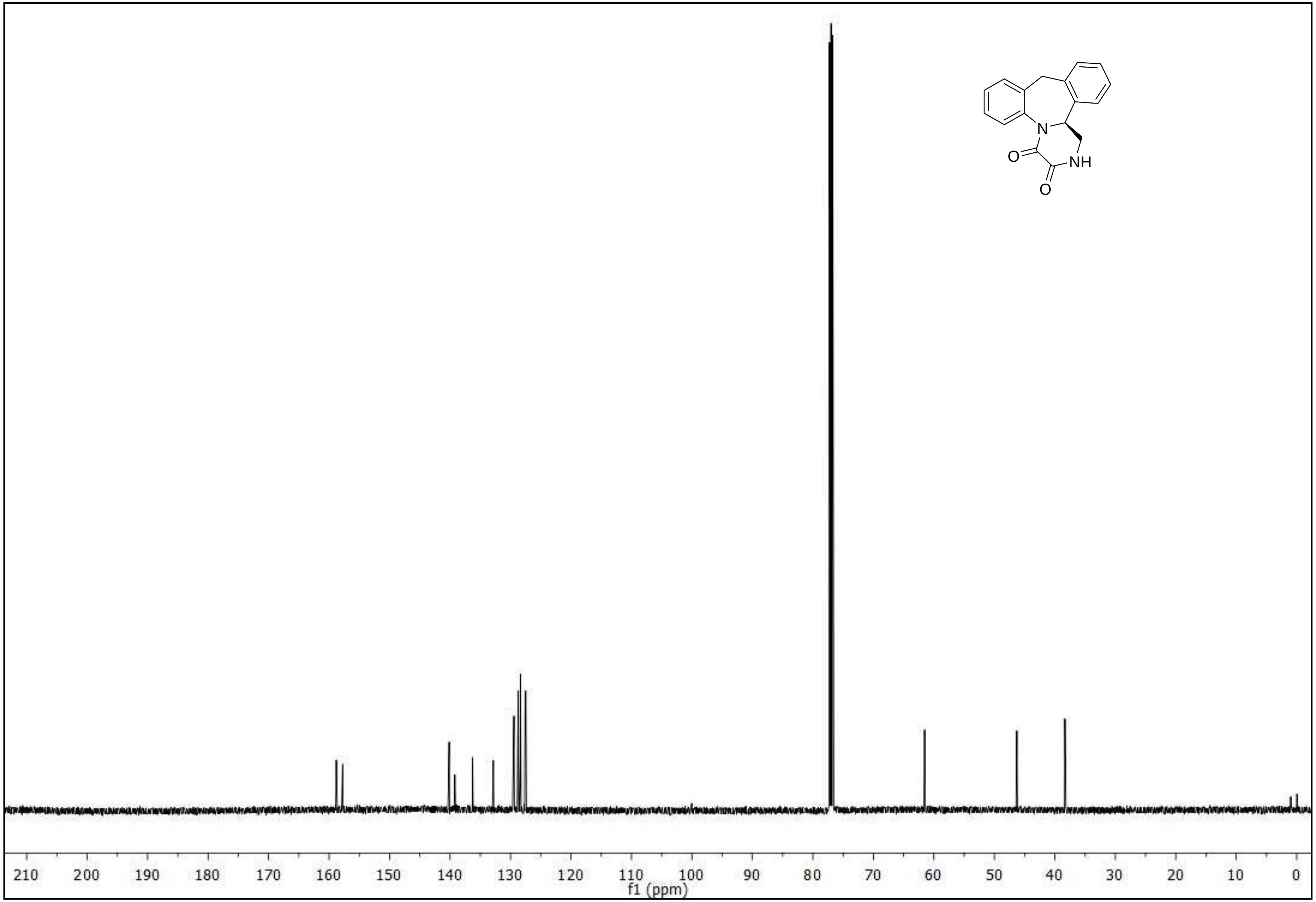
N
O
NH
O
210
200
190
180
170
160
150
140
130
120
110
100
90
80
70
60
50
40
30
20
10
0
f1 (ppm)

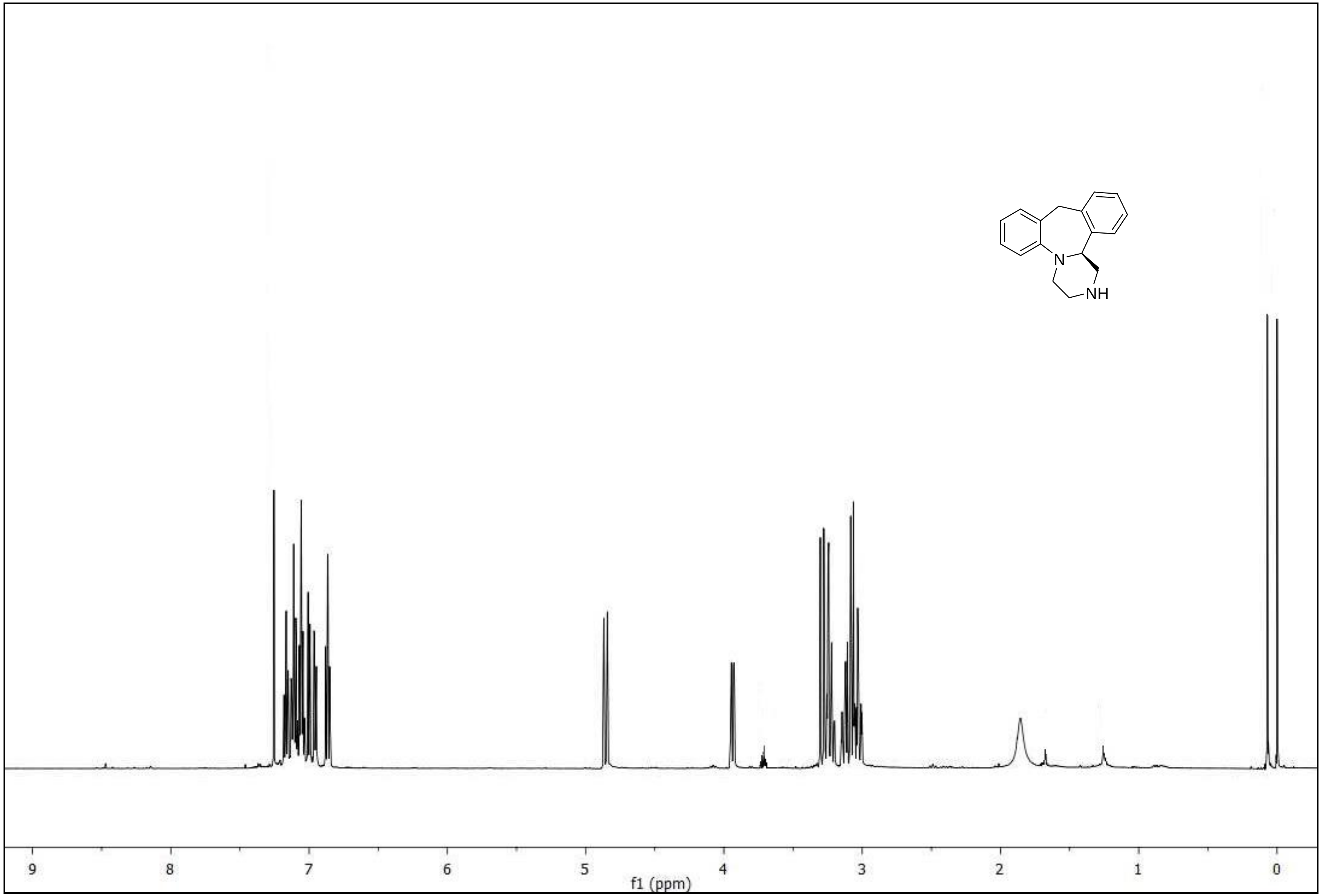
N
NH
9
8
7
6
5
4
3
2
1
0
f1 (ppm)

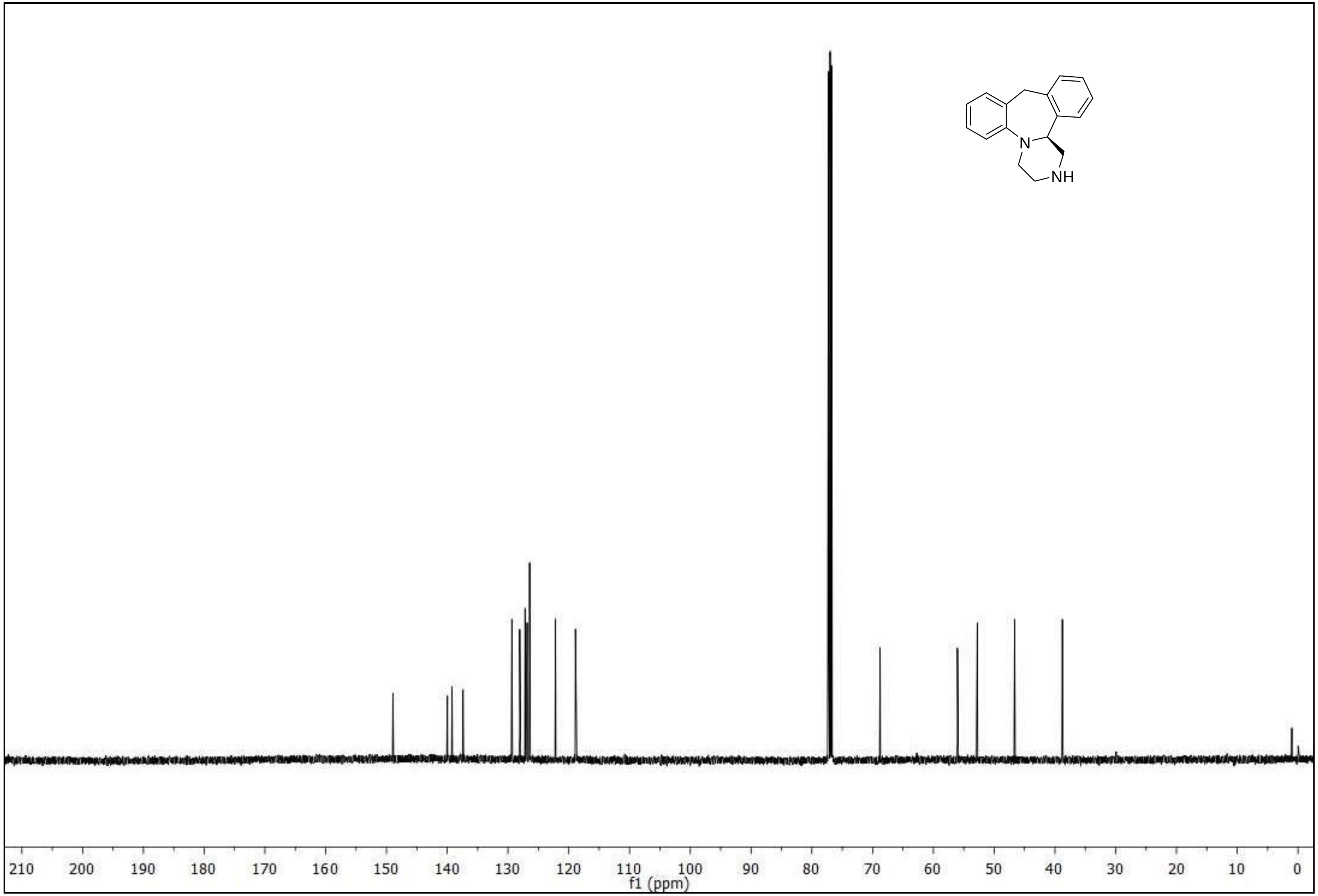
N
NH
210
200
190
180
170
160
150
140
130
120
110
100
90
80
70
60
50
40
30
20
10
0
f1 (ppm)

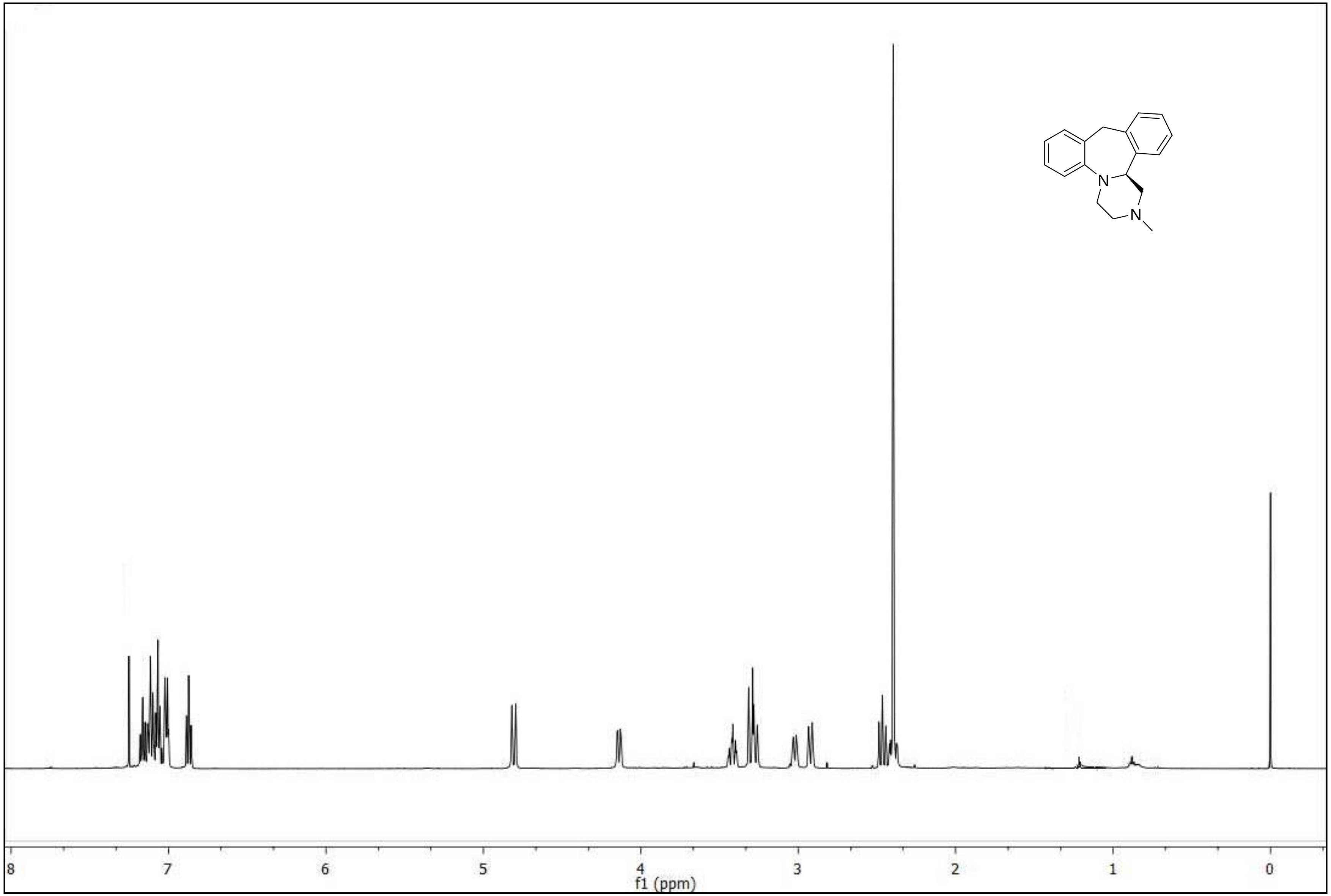

N
N
8
7
6
5
4
3
2
1
0
f1 (ppm)

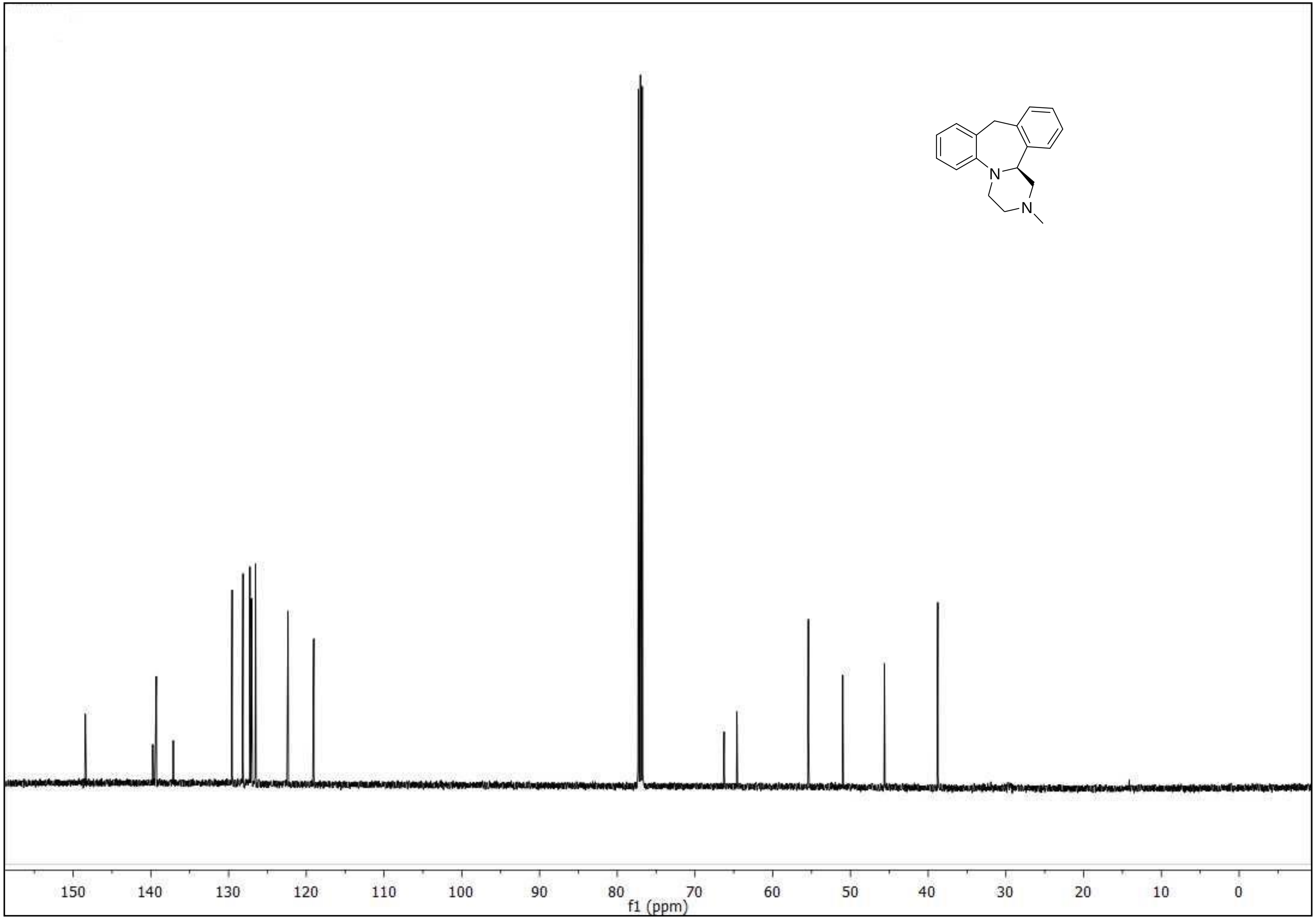

N
N
150
140
130
120
110
100
90
80
70
60
50
40
30
20
10
0
f1 (ppm)

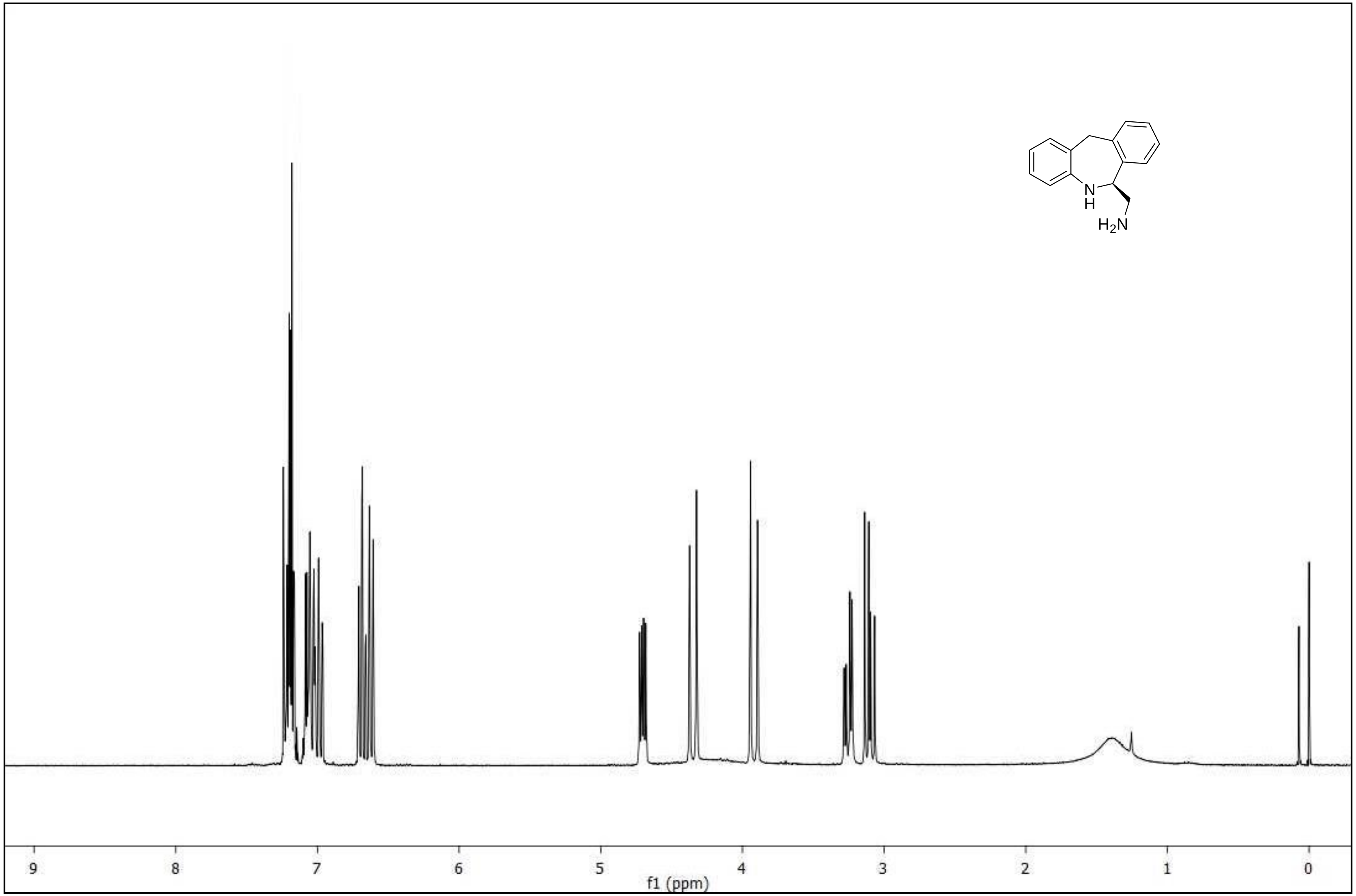

H
N
H2N
9
8
7
6
5
4
3
2
1
0
f1 (ppm)

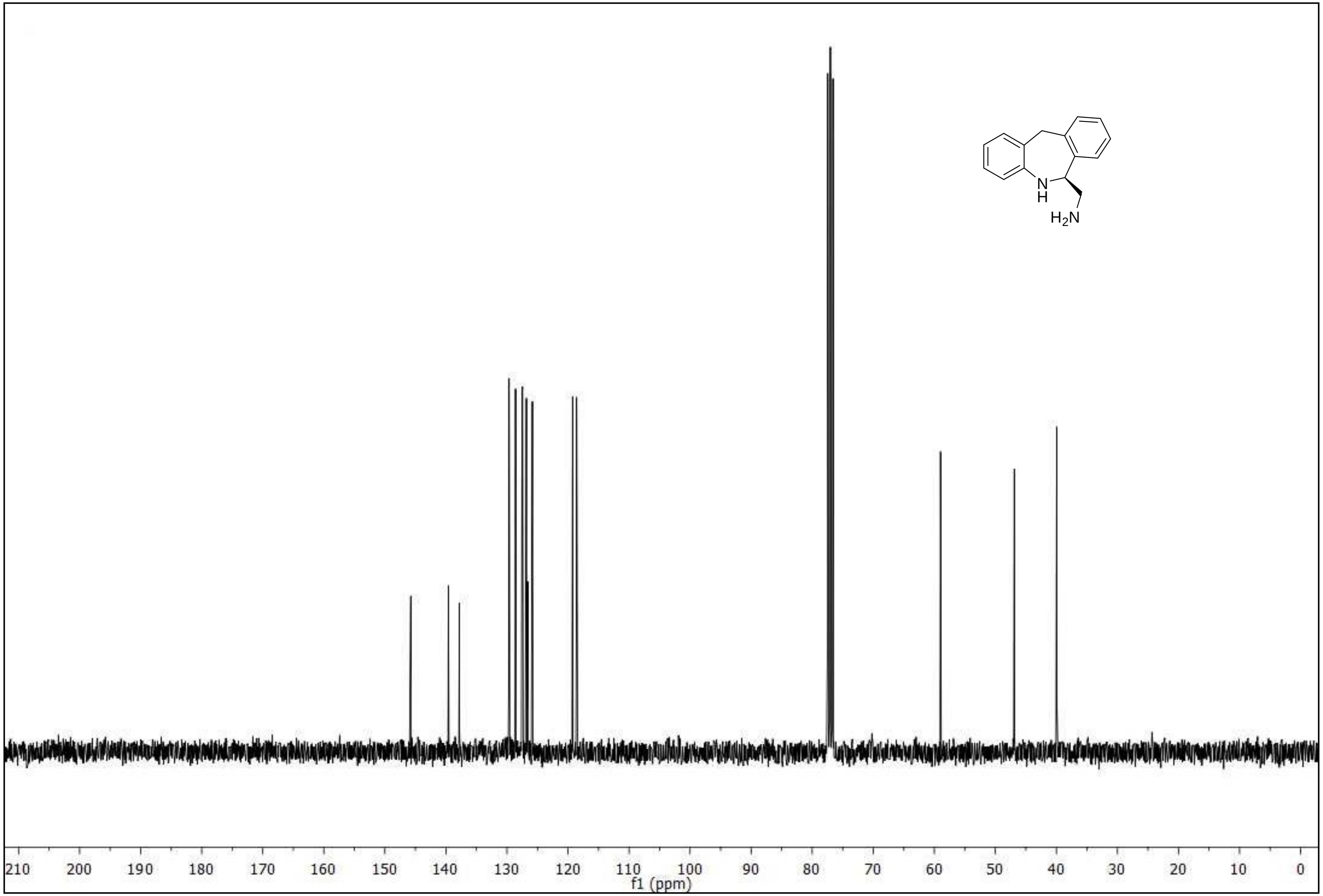
N
H
H2N
210
200
190
180
170
160
150
140
130
120
110
100
90
80
70
60
50
40
30
20
10
0
f1 (ppm)

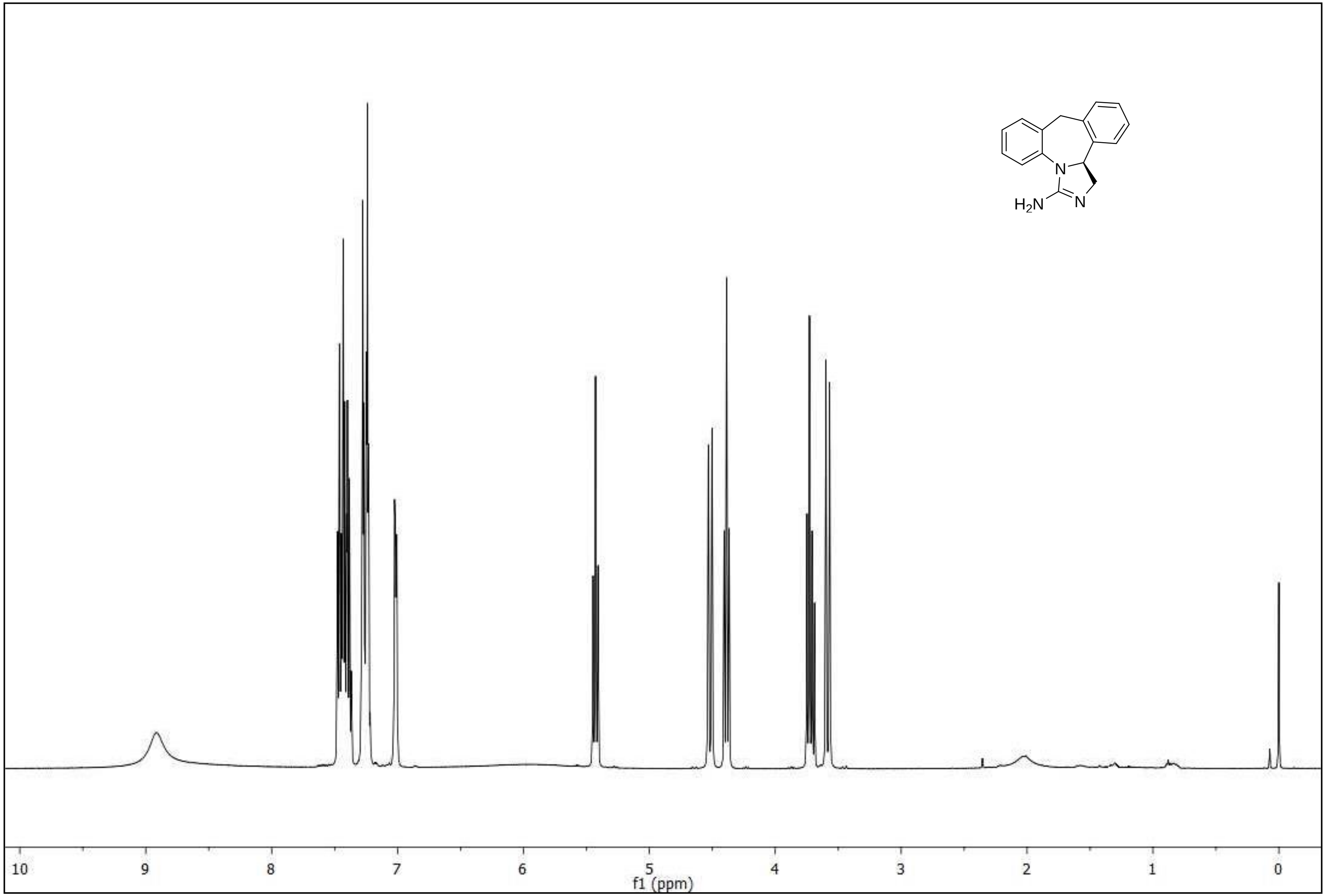

H2N
N
N
10
9
8
7
6
5
4
3
2
1
0
f1 (ppm)

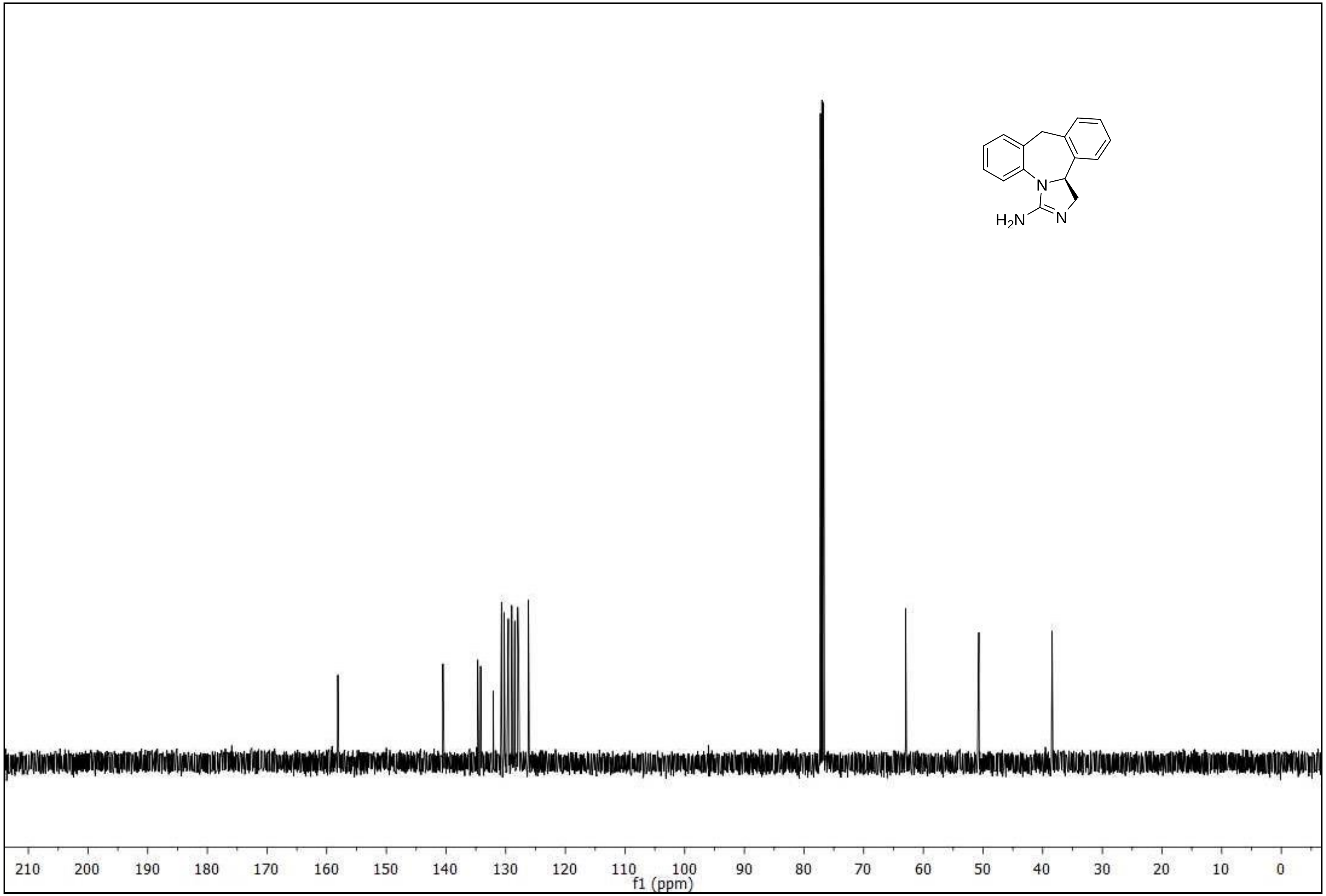

H2N
N
N
210
200
190
180
170
160
150
140
130
120
110
100
90
80
70
60
50
40
30
20
10
0
f1 (ppm)
